# The Current Evidence Levels for Biofeedback and Neurofeedback Interventions in Treating Depression: A Narrative Review

**DOI:** 10.1155/2021/8878857

**Published:** 2021-02-04

**Authors:** Mikhail Ye. Melnikov

**Affiliations:** Biofeedback Computer Systems Laboratory, Institute of Molecular Biology and Biophysics, Federal Research Centre of Fundamental and Translational Medicine, 630060 Novosibirsk, Russia

## Abstract

This article is aimed at showing the current level of evidence for the usage of biofeedback and neurofeedback to treat depression along with a detailed review of the studies in the field and a discussion of rationale for utilizing each protocol. La Vaque et al. criteria endorsed by the Association for Applied Psychophysiology and Biofeedback and International Society for Neuroregulation & Research were accepted as a means of study evaluation. Heart rate variability (HRV) biofeedback was found to be moderately supportable as a treatment of MDD while outcome measure was a subjective questionnaire like Beck Depression Inventory (level 3/5, “probably efficacious”). Electroencephalographic (EEG) neurofeedback protocols, namely, alpha-theta, alpha, and sensorimotor rhythm upregulation, all qualify for level 2/5, “possibly efficacious.” Frontal alpha asymmetry protocol also received limited evidence of effect in depression (level 2/5, “possibly efficacious”). Finally, the two most influential real-time functional magnetic resonance imaging (rt-fMRI) neurofeedback protocols targeting the amygdala and the frontal cortices both demonstrate some effectiveness, though lack replications (level 2/5, “possibly efficacious”). Thus, neurofeedback specifically targeting depression is moderately supported by existing studies (all fit level 2/5, “possibly efficacious”). The greatest complication preventing certain protocols from reaching higher evidence levels is a relatively high number of uncontrolled studies and an absence of accurate replications arising from the heterogeneity in protocol details, course lengths, measures of improvement, control conditions, and sample characteristics.

## 1. Introduction

Over the approximately 60-year-long history of neurofeedback, depression has received much attention from researchers and clinicians. A number of electroencephalographic (EEG) and real-time functional magnetic resonance imaging (rt-fMRI) protocols were developed alongside biofeedback approaches. Over 100 studies are dedicated to depression and view it either as a mental disorder or as a subclinical personality trait or emotional state, and nearly 25 trials deal with clinical depression per se figuring out biofeedback or neurofeedback utility as an adjunct treatment. A number of reviews and meta-analyses were published to date summarizing data on effectiveness of certain modalities and protocols in treating depression [1–8], with most of them assessed effect sizes and other measures of biofeedback- or neurofeedback-related benefits. Only few of them were devoted to evidence levels that are a gross evaluation of methodology implemented in the studies on the problem and reflect the degree of our current confidence in effectiveness of existing biofeedback and neurofeedback practices for depression treatment [9, 10]. A criteria list developed by La Vaque et al. [11] and endorsed by the Association for Applied Psychophysiology and Biofeedback (AAPB) and International Society for Neuroregulation & Research (ISNR) comprises five levels from “not empirically supported” to “efficacious and specific” with each next stage presenting additional demands to study quality and/or quantity (see Table 1). This model is relatively simple and operationalized, so it was chosen as a basis for the current review.

To our knowledge, these criteria have been previously adopted in two reviews. First, Larsen and Sherlin [9] estimated efficacy levels of depression neurofeedback on AAPB criteria as 2/5 “possibly efficacious.” In the most recent review based on these criteria, Shaffer and Zerr [10] in *Evidence-Based Practice in Biofeedback and Neurofeedback* outlined the current evidence level for widespread biofeedback and neurofeedback protocols including ones of rt-fMRI neurofeedback. The authors considered three protocols as 4/5 “efficacious,” namely, heart rate variability (HRV) biofeedback, frontal alpha asymmetry EEG protocol, and fMRI neurofeedback. While enough support for this level was shown for HRV biofeedback, other two approaches possibly were overestimated, which could stem from overly permissive interpretation of the criteria and mixing together different rt-fMRI protocols.

This article reviews studies of depression treatment with any biofeedback or neurofeedback approach and does not differentiate between modalities which are more likely to have a specific impact on depression (fMRI, HRV, and EEG frontal alpha asymmetry) and ones that are assumed to influence depression indirectly or nonspecifically (other EEG protocols, EMG, and thermal), though a brief discussion of models explaining the effect of each modality and protocol on depression is introduced. To account for the heterogeneity of depression, separate subsections were formed for major depression and other depressive disorders. The structure with sections illustrating modalities and protocols and subsections devoted to different levels of disorder severity brings clarity and was adopted from the review by Shaffer and Zerr [10].

Published studies allocated to each subsection were screened using criteria shown in Table 1. A liberal approach to questionable cases was chosen. The 1st level of evidence was taken as minimal possible even if no studies supporting presence of positive effect of protocol on depression were found, for anecdotal evidence may be unpublished. Any significant positive change of outcome measure within group (pre- vs. posttreatment) was accepted as matching “demonstrates efficacy” criterion (level 1) without necessarily having to meet minimal clinically important values for depression (HAM‐D < 5 for full remission, HAM‐D < 9 for partial remission, and 25-35% or 5-6 points' reduction for minimal clinically relevant changes [12]).

We found no studies omitted outcome measure (level 2). Outcome measures were classified to clinical (C—scales based on psychiatrist's expertise, namely, Montgomery-Asberg Depression Rating Scale (MADRS) and Hamilton Depression Rating Scale (HAM-D)), psychological-1 (P1—widely used psychological assessment of depression, namely, Beck Depression Inventory (BDI), Zung Self-Rating Depression Scale (ZSRDS), Hospital Anxiety and Depression Scale (HADS), and depression scale of Symptom CheckList-90 (SCL-90)), psychological-2 (P2—other depression tests or depression scales of personality tests), mood (M—state-measuring instruments for mood like Profile of Mood States (PoMS) and Positive and Negative Affect Schedule (PANAS)), and cognitive (Cog—instruments measuring cognitive features related to depression, like attention bias). If a significant within-group difference was demonstrated, the study was treated as adequately statistically powered (level 2) irrespective of group size.

Multiple studies' criterion (level 3) was treated as at least two studies showing within-group positive change related to the same protocol in the similar population on outcome measures belonging to the same group. “Noninferior to treatment as usual” (TAU) criterion (level 4) was met once study compared sole biofeedback against established treatment, not when biofeedback added to usual care was compared to TAU alone. “Superior” and “noninferior” (level 4, 5) were operationalized in terms of presence or absence of significant intergroup difference at posttreatment or follow-up point, regardless of effect sizes and clinical importance. The “inclusion criteria” (level 4) were understood as indicating certain diagnosis or range on a standardized assessment scale and brief demographical summary.

From the “data analysis” point of view (for level 4), a study was accepted if no major misuses of statistics were evident and if statistics were understandable from the article text, not necessarily from the special section but also from the tables, coefficients mentioned in results, etc. Absence of data on effect sizes, adjustments for multiple comparisons, and widespread, “traditional” statistical misuses, like treating scores of psychological assessments as an interval scale instead of ordinal or not examining distributions and sphericity, were not considered serious violations. The “2+ independent studies” criterion (level 4) required at least two articles with no common authors or authors' affiliations both fulfilling other requirements of levels 1-4. For some ambiguous cases, a Yes/No (Y/N) mark was used indicating that the study partly suits a criterion, e.g., for crossover studies, presence of a control group is marked as Y/N.

The characteristic features of the current review are the following: (1) discusses evidence levels rather than effect sizes and uses the La Vaque et al. [11] criteria set; (2) focuses on depression only, not anxiety or other psychopathology which allows for a detailed discussion of the studies; (3) features a narrative structure, not systematic, which allows for combining descriptions of certain studies and rationale and action models behind protocols; (4) comprises data on any modality and protocol; (5) differentiates between protocols and clinical populations to prevent overgeneralization of the findings; (6) excludes case studies and papers documenting groups of extremely small size; (7) pays special attention to relatively novel rt-fMRI neurofeedback modality.

The two main questions the article is aimed at answering are the following. (1) What are the evidence levels of existing biofeedback and neurofeedback protocols for treating depression in certain populations? In other words, can we recommend any biofeedback or neurofeedback approach to cure depression? (2) What are the methodological issues preventing the studies of these protocols from getting “upstairs”?

## 2. General Biofeedback and Neurofeedback Mechanisms

Biofeedback is a treatment and research technology based on self-regulatory abilities of a patient or study participant. Biofeedback refers to learning to voluntarily change a measurable biological parameter which normally cannot be regulated consciously, but may become controllable through exercise. Target signal (e.g., related to a symptom of disorder) is measured and fed back to a participant enabling them to find their own strategy to control this signal and then adjust this strategy to master self-regulatory performance which then may be generalized to everyday life. Thus, biofeedback and neurofeedback (biofeedback targeted to brain signals) may be utilized as treatments or as research approaches aimed at testing hypotheses about causal relations between localized neural activity and symptoms [13]. Biofeedback interfaces are usually portable and easy to use so they can be leased to a patient for the treatment period for home practice, and some simple interfaces are affordable for purchase and daily personal use. Thus, one crucial advantage of biofeedback is that it enables patient self-help without binding to a specific location or specialist, which improves treatment accessibility and prevents the development of stigma [14].

Biofeedback is well known as a treatment option for mental disorders, e.g., according to Weinman et al. [15], 80% of patients with generalized anxiety disorder have heard something about biofeedback, and 60% have more certain knowledge. Self-regulation treatment may be expensive and is not currently covered by insurance [16]. However, Schoenberg and David [17] treat biofeedback as a common, especially in the US, and cost-effective (cost-benefit ratio from 1 : 2 to 1 : 5) nonpharmacological approach to severe depression, while only 1.5% studies of depression treatment involve this intervention.

From the perspective of general mechanism, biofeedback and neurofeedback are frequently treated as based on operational conditioning, which means that learning to control some biological signal is acquired via multiple trials and behaviour adjustments in order to achieve more frequent reinforcement (positive feedback scores) [18, 19]. Biofeedback is based on two major cognitive skills: ability to identify the rewarded state (internal feedback) and ability to adjust the current state in the desired direction. So biofeedback may be understood as promoting lacking information on performance or result of the cognitive effort and helping one to fine-tune his/her intrinsic feedback [18]. While feedback is presented explicitly, which is the case in the majority of clinical applications, such learning should be referred to as model based, meaning that the participant intentionally seeks a reward, leading to prevalence of top-down regulation operating with focus of attention and working memory content. In cases of implicit feedback, model-free learning takes place with prominent bottom-up processes [18]. Dual-process theory implies that conscious and unconscious learning processes (top-down and bottom-up) are present at the same time. So the trainee gains both some overt strategies and covert “intuition” which cannot be disclosed [18, 19]. On the first stage, conscious compounds dominate, and when skill is generally learned, unconscious learning plays a major role in its adjustment [18].

Some authors stress the role of strategies, e.g., Garnefski and Kraaij [20] consider biofeedback as an approach to validating beneficial (positive reappraisal) and dysfunctional (catastrophizing, rumination, and self-blame) emotional coping strategies. MacDuffie et al. [21] recommend single sessions of neurofeedback as a means to show the neural effects of some cognitive strategies learned in the course of cognitive behavioural therapy. However, in some cases, feedback may be fully implicit while still effective [18]. Birbaumer et al. [22], in their review with special attention to animal studies, defined neurofeedback as a skill learning technique, which primarily relies on unconscious mechanisms and is similar to the acquisition of motor skills. Paret et al. [19] implied that, while suggested strategies are not required for neurofeedback and may even hamper learning and performance in general, it may be helpful in the case of emotional regulation, for some cognitive strategies are known to be more appropriate compared to others. In some cases, including depression, instructions may prevent harmful strategies undistinguishable from useful in terms of their impact on brain activity [23]. Left amygdala fMRI activation used as neurofeedback target in a few studies may be achieved through self-induced sadness while feedback is being presented, and amygdala response is correlated with self-rating of sadness [24]. Left-sided frontal asymmetry, the most reputable EEG target for neurofeedback in depression, may be driven by anger as well as by positive emotions [25]. Thus, the role of strategies remains unclear, with some publications showing that explicit strategies are not necessary for learning, some arguing for and some against suggested strategies.

Mechanisms and dynamics of accumulation effects from session to session and their fade out posttreatment are not fully understood. “Growth through utilization” mechanism is sometimes mentioned to address biofeedback-related changes during the course [9]. Dynamics of neurofeedback-related learning is rather heterogeneous and frequently assumed to be nonlinear. Some subjects achieve their maxima after the first session, while others need far more training time [19, 26]. Evidence that corroborates sustainability of rt-fMRI NFB learning and evidence that denies it is of nearly the same argumentative value with some studies published supporting each possibility [23].

From the neuroscience point of view, neurofeedback learning activates neuroplasticity mechanisms equivalent to those related to acquiring other skills and uses natural gradients of brain functional states. The difference lies in the presence of clear and direct feedback leading to more rapid and less ambiguous learning and underlying plastic changes. According to Othmer et al. [27], the key element of neurofeedback is linking the feedback to brain activity, and once the link is established, the target activity is being regulated in an automated fashion by means of brain homeostatic and dynamic mechanisms. Paret et al. [19] discuss and in general approve the idea of the presence of at least three nonspecific neurofeedback-related brain systems expressed by Sitaram et al. [26]: first, the system observing and processing feedback; second, the learning system; and third, the reward system. A whole “neurofeedback system” according to Paret et al. [19] comprises the lateral prefrontal, posterior parietal, anterior cingulate, and insular cortex along with lateral thalamus or, according to Emmert et al. [28], the prefrontal, temporal-parietal, temporal-occipital, anterior cingulate, and anterior insular cortex along with striatum, which are related to motivation, body awareness, multimodal sensory integration, and cognitive control. Sham (false) rt-fMRI NFB condition is known to activate the anterior cingulate, insula, motor, and prefrontal cortices [23]. Birbaumer et al. [22] specifically stress the basal ganglia impact on neurofeedback success, while the role of other motor system components is considered to be less evident.

Aside from increase in target biological signal biofeedback, experience may influence some psychological traits such as alexithymia [29, 30] and promote improvements through these changes. Peniston et al. [31] and Klee and Meyer [32] discussed an impact on a learned helplessness as a possible factor of depression improvement with biofeedback. Biofeedback teaches patients that they can avoid aversive stimuli, which is controversial to a learned helplessness mindset and is in line with a self-efficacy mindset. Biofeedback- or neurofeedback-related gains in self-efficacy or related variables were mentioned in some studies [33–37]. Mehler et al. [36] suggested that the rewarding nature of neurofeedback may be an explanation of its effects. Patients in frontal alpha asymmetry course were shown to experience self-competition and report feelings of competence and pride or frustration depending on their performance [38]. EMG biofeedback trainees tend to link their improvements with acquired self-control or with the efforts done [15]. Linden [29] stressed the gaming component as a nonspecific advantage of neurofeedback making it more engaging and attractive compared to straightforward talking psychotherapy. Biofeedback and neurofeedback are also frequently treated as interventions promoting relaxation and combating stress, which may to some degree explain their effects in depression [39]. For instance, patients who underwent HRV biofeedback course while being directly asked about key factors of their improvement name better interoception, enhanced stress regulation, and more mental stability [40].

Participants themselves are not all equally sensitive to self-regulation learning. Attention span, concentration ability, and motivation are demonstrated to be related to neurofeedback success. Mood also plays a role, especially in emotionally related tasks and conditions, while the influence of personality factors lacks strong evidence [41]. In some cases, healthy volunteers are less capable to achieve brain activity self-regulation than depressed ones, which may be due to lower motivation [42]. In depressed participants, the level of motivation may decrease along the course with a kind of “fatigue” [42], which is an argument against the long-as-possible courses. It seems logical that the deficit in approach motivation and diminished reward sensitivity lead to decreased performance of depressed patients in neurofeedback as a reward-based technique; however, existing data supports the ability of neurofeedback to improve approach motivation even in seriously depressed participants [43].

Biofeedback is frequently claimed to be free of side effects [16] or at least to have less prominent side effects due to the fact that intervention is usually band- and lead-specific in EEG or region of interest- (RoI-) specific in fMRI and has a local action [13]. Researchers who record and report side effects (e.g., [44, 45]) mostly find ones indicating discomfort rather than real harm, namely, dry mouth, headache, dizziness, and fatigue. Hawkinson et al. [46] reported that adverse effects in rt-fMRI training occur as frequently as in usual fMRI scanning, namely, less than 10%. These effects are not serious and do not influence withdrawal from the training course. However, Walker et al. [8] and Larsen and Sherlin [9] argue that side effects of neurofeedback are underestimated and may include agitation, cognitive interference, anxiety, irritability, and aggression.

Studies in the field of biofeedback and neurofeedback have been frequently criticized for poor methodology, while some well-controlled studies led to negative results. Thibault et al. [23] in their review claimed that the most methodologically robust studies of EEG neurofeedback did not support its superiority over sham neurofeedback. The authors mentioned that evidence on EEG neurofeedback in depression is still insufficient. Most studies that reported behavioural effects of neurofeedback failed to demonstrate their specificity [23]. Arns et al. [18] in their review concluded that both EEG and fMRI neurofeedback research were not mature enough to consider them as psychiatric treatment except EEG protocol for ADHD. Methodology was mentioned as a key reason for skepticism, namely, small samples, lack of randomized or blind design, inappropriate control conditions, and low quality of neurofeedback itself. Thibault et al. [7] points to the necessity of high research standards for neurofeedback development, namely, double-blind designs with adequate placebo condition. A small comment by Thibault et al. [47] underscored that neurofeedback environment is an extremely placebogenic one, and neurofeedback placebo effect may surpass specific effects of some treatments. Review of recent placebo-controlled neurofeedback trials in ADHD led authors to a conclusion that neurofeedback lacks specific components, shows no evidence of self-regulation learning, and acts mainly as a placebo [48]. However, Pigott et al. [49] responded to this criticism pointing to the fact that all papers reviewed in [48] featured positive feedback 80% of session time which prevents operant conditioning. The recently proposed CRED-nf standard of research reporting with substantial implications for designs [50] may help improve the quality of evidence originating from the contemporary studies in the neurofeedback field.

Subsequent sections will observe published studies of biofeedback depression treatment in all major modalities, focusing on each existing protocol. The rationale for the usage of each protocol will be given, followed by results of the corresponding studies, concluding with a table demonstrating the current evidence level.

## 3. Heart Rate Variability (HRV) and Respiratory Sinus Arrhythmia (RSA) Biofeedback

HRV arises from the fluctuations of heart rate related to inhalation and exhalation and mediated by vagus nerve activity [51]. Thus, HRV reflects cardiovascular ability to adapt to sympathetic influences, synchronicity between respiration and cardiac activity, accompanied by high amplitude of cardiac oscillations and may be treated as an index of cardiovascular health [51, 52]. In frequency domain, high frequencies are related to parasympathetic activity, while low frequencies reflect sympathetic activity and baroreflex [52].

HRV is known to be linked to mood. Appelhans and Luecken [53] in their review approved of the usage of HRV as an emotional regulation index taking into account both excitation and inhibition (sympathetic and parasympathetic influences). Higher HRV is observed in those who are more persistent in solving complex problems while intensive self-control efforts are required [54]. A shift from parasympathetic to sympathetic autonomic activity resulting in cardiac dysautonomia is frequently found in depression [55]. Karavidas [56] stated that depression is associated with a decreased HRV. This link is related to a decreased vagal tone (activity of the vagus nerve) and augmented sympathetic activity and may be interpreted as a rigidity of emotional regulation [51, 57, 58]. Kemp et al. [59] showed that HRV deficit in MDD is related to a condition, rather than to medication, and is most pronounced in MDD comorbidity with generalized anxiety disorder. Effectiveness of the antidepressant medication and psychotherapy may be compromised by the fact that HRV does not recover during the treatment courses, which means that while patients improve on symptoms they still stay stress-prone which negatively influences final outcomes and retention [57].

HRV and related modalities of biofeedback are frequently used to treat depression as a primary condition. According to Blase et al. [1], HRV biofeedback improves depression especially when used in combination with psychotherapy: in reviewed studies, patients of the biofeedback group reached up to 78% reduction on BDI scores, while controls only reached up to 48%. A recent meta-analysis by Lehrer et al. [2] found *g* = −0.25 for depression improvement in 21 randomized controlled studies of HRV biofeedback and *g* = −0.72 for 5 of them specifically targeted depression. Training effect is generally greater in studies with a passive control group compared to active and practically independent on course length [2], which may raise questions as to the specificity of the effect.

HRV training typically requires adjusting breathing to a resonant frequency which is approximately 0.1 Hz with some individual variance to achieve maximum RSA and HRV that counters hyperventilation and increases parasympathetic activity [2, 56]. The rationale is that, by increasing HRV and parasympathetic activity via biofeedback training, patients will gain better control of their emotional reactions, which consequently will lead to a reduction of depression symptoms. The exact amplitude of HRV changes in biofeedback is mostly dependent on participants' age (lower in older trainees) and unrelated to behavioural or clinical changes [2], so acquiring the skill seems more important than the degree of HRV modulation.

Biofeedback is known to be effective in lowering systolic and diastolic blood pressure to some extent, so it may facilitate production of nitric oxide (vasodilator). Nitric oxide-associated relaxation effects are involved in the improvement of symptoms of various disorders including depression [60], and this can be even more pronounced in the case of HRV biofeedback. HRV biofeedback added to psychotherapy may produce benefits by reducing stress and withdrawal rates at the early stages of treatment [57].

A psychological effect also was proposed, namely, meditation with shifting attention focus to breathing. According to Servant et al. [58], HRV biofeedback with paced breathing, relaxation, or meditation strategy may be effective in upregulating HRV; however, the relative impact of these strategies is unknown. On a behavioural level, depressed patients exhibit a reduced accuracy of their own heartbeat perception [61] that may be reversed during HRV biofeedback; however, it is unclear whether this change in interoception influences mood.

Lehrer and Gevirtz [62] in their review discussed some possible neural models of HRV biofeedback action. A few of them mentioned also in [56, 63] are relevant to depression. The first one is related to baroreflex gain, since baroreflex is mediated by nucleus tractus solitarius of the brainstem, which has connections to the amygdala and insula. Second (and the most important for emotional disturbances) is related to diaphragmatic breathing with stimulation of the subdiaphragmatic vagus nerve afferent pathways that are connected to some emotional brain areas including the insula, the amygdala, and the orbital frontal area. Vagus efferent pathways may be modulated via an “accentuated antagonism” mechanism by blocking some sympathetic output to produce parasympathetic nonspecific relaxation.

The rationale for the usage of heart coherence protocol is mainly the same as for HRV biofeedback. RSA or cardiac coherence is related to nearly equal interbeat intervals [58] and is a marker of sympathetic-parasympathetic balance that is not fully dependent on HRV [52], while low-frequency (0.05-0.15 Hz) powers are typically being upregulated with RSA maximization [56]. Some evidence exists for higher coherence scores during relaxed state and positive emotions and lower in negative emotional states and in psychological stress. Respiratory trainings including resonant frequency breathing are supposed to be instrumental in changing heart coherence scores [52].

Patients with cardiac complications are likely to decrease their depression estimates after a course of HRV training (see [64] for a review, [65–67] for examples, and [52] for contradicting results). Few trials in noncardiac conditions are published with the strongest evidence for depression score reduction in chronic fatigue syndrome [40] and PTSD [68, 69]. Three studies showed no HRV biofeedback effect on depression in substance use disorder [70–72]. Studies in normal samples were mostly focused on participants with elevated stress or anxiety. Some of them showed no improvement on subjective depression [14, 73–75], while others [74, 76, 77] demonstrated some benefits, though failed to prove their specificity.

It is worth noting that a few studies support self-practice in HRV biofeedback in subclinical depression. Participants with high Patient Health Questionnaire score, though no formal diagnosis of depression, were nearly twice more likely to respond to mobile phone app-based depression self-help course while HRV biofeedback component was included there [78]. A nonrandomized controlled study showed that daily home practice in HRV biofeedback increases SDNN and decreases Edinburgh Postnatal Depression Scale score [79].

### 3.1. Major Depressive Disorder (MDD)

A few studies of HRV biofeedback in MDD have been published to date. First, in an uncontrolled trial with 8 patients, in a course of 10 weekly sessions (additional 2 × 20-minute daily home practice was encouraged) of HRV biofeedback using resonant frequency breathing strategy, there were reduced depression scores of BDI-II and HAM-D (*d* = 3.6). The first clinical improvement occurred simultaneously with augmenting of the standard deviation of normal cardiac interbeat intervals (SDNN), which then unexpectedly returned to the initial values, meaning that positive effects did not last. Physiological changes comprised ones of low-frequency amplitudes and SDNN [80]. The author announced a sham-controlled replication; the preliminary (*N* = 5 in both groups) results of which suggested improvement on BDI-II scores and within-session improvement on low-frequency power [56]; however, we could not find the ultimate results published.

A randomized controlled study [51, 57] involved 10 female students with MDD who received five 30- to 45-minute sessions of HRV biofeedback with diaphragmatic and resonant frequency breathing along with recommendations for daily home practice in addition to acceptance and commitment psychotherapy. Compared to the group that received psychotherapy only, the biofeedback group improved better on BDI-II scores (partial *η*^2^ = 0.3). HRV features of depressed participants at baseline were equivalent to the ones of healthy volunteers. However, the experimental group changed SDNN, high-frequency power, and high/low-frequency ratio during the course, and it had been demonstrated that SDNN changes drove depression reduction. fMRI resting state functional connectivity modulations between the amygdala, the hippocampus, and the anterior cingulate were expected; however, no significant improvement was achieved.

In one more study [81], adult depressed patients who received four HRV biofeedback (real or sham—“wrong” yet harmless for resonant breathing frequencies) 20-minute sessions once a week and then were encouraged to continue practicing at home improved their depression estimates (HAM-D and BDI-II) by the end of the course and by the 3- and 6-week posttraining follow-ups. A variety of other psychological metrics improved, such as anxiety, hopelessness, suicide risk, and some domains of social functioning. No significant differences between the real and sham groups were found (ANOVA group × time), which may be related to small sample size (11 participants finished training in total, 6 in the experimental group).

Lastly, in a recent study [82], 24 participants with a primary MDD diagnosis and additional sleep problems underwent six weekly one-hour sessions of resonant frequency breathing HRV biofeedback in addition to usual medical care. Training session included muscle relaxation, diaphragmatic breathing, paced breathing, pursed lip breathing, and some psychoeducation. 10 minutes of home exercises daily were recommended. The biofeedback group had decreased BDI-II estimates, both cognitive (*η*^2^ = 0.43) and somatic (*η*^2^ = 0.31) subscales, and also anxiety and presleep arousal, while gender- and age-matched controls who received standard care only did not. Gains in BDI-II were also evident at one-month follow-up. SDNN, LF, and total power increased in trainees.

### 3.2. Depressive Disorders Other Than MDD

In a systematic review of six randomized controlled trials involving HRV biofeedback in patients with affective and anxiety disorders, HRV biofeedback was shown to be more efficient than no treatment condition both at posttreatment and at one- or two-month follow-up [1].

In addition to this, in one trial, a group of 14 patients with different depression conditions improved on BDI scores, diminished heart rate, and increased HRV after six triple-a-week 25-minute HRV biofeedback sessions with paced breathing guide. A group of healthy volunteers underwent the same course, and another control group was present as a no treatment condition, while no control group of depressed patients was involved. It is noteworthy that healthy people did not benefit from the same protocol [83].

In another study, a group of 15 patients with postpartum depression received two 30- to 60-minute sessions of HRV biofeedback with deep, abdominal or diaphragmatic breathing strategy in order to cope with anxiety, which is typical for postpartum depression. Posttreatment participants improved on anxiety and well-being, while perinatal depression score was measured only at baseline and included in the analysis just as a covariate. The majority of participants approved continuing using breathing techniques once a week or more frequently and usefulness of this strategy [84].

A sample of Thai depressed inpatients of mean age of 76 who received two 30-minute HRV biofeedback sessions a week, for five weeks, reduced their depression scores (Depressive Cognition Scale and Thai Geriatric Depression Scale), and controls (morning exercises and social activities) did not. Each group included 50 patients, and groups did not differ at baseline [85].

Results from this section are summarized in Table 2.

### 3.3. Conclusion

Aside from two studies [82, 85], HRV biofeedback training for depressive disorder was assessed in small-sample studies, partly uncontrolled. Two controlled studies of sufficient size were done in specific depression subpopulations, namely, depression in the elderly and depression with comorbid sleep disturbance, so these results may not necessarily be projected onto the whole population of depressed patients. Since some controlled studies showed superiority over no treatment condition, subsequent controlled studies involving samples of sufficient size may increase the credibility of the protocol.

Note that training courses were relatively short, ranging from 2 to 10 sessions, most frequently 4 to 6, with suggestion to continue home practice. This makes the intervention time- and cost-effective on the one hand and raises the question of whether patients would improve more if they underwent more sessions on the other hand.

Importantly, a few studies in MDD population demonstrate concurrent changes in depression and HRV parameters, which is a strong support for biofeedback action specificity. SDNN change may be a relatively reliable marker of depression improvement. Some studies also mention increases of low-frequency power and high-frequency power and decreases of heart rate that possibly indicate different biofeedback action mechanisms.

Thus, HRV biofeedback is considered by some independent groups to be effective in clinical depression; however, specific effects were demonstrated compared to no treatment only, not against sham biofeedback. With two relatively similar studies with some differences in the sample details [57, 82], we would recommend assigning level 3 (“probably efficacious”) of evidence to HRV biofeedback courses with additional home practice in patients who suffer from MDD as a primary complication. A well-established psychological scale (e.g., BDI-II) must be used to measure outcomes to meet level 3 criteria.

## 4. Electroencephalographic (EEG) Neurofeedback

Neurofeedback is a biofeedback that targets brain signals, including such submodalities as surface EEG, live *Z* scores, low-resolution electromagnetic tomography (LORETA), slow cortical potentials (SCP), low energy neurofeedback system (LENS), magnetoencephalography (MEG), fMRI, and functional near-infrared spectroscopy (fNIRS) [16]. Neurofeedback is believed to induce a long-term potentiation and thus facilitate synaptogenesis. On the macroscopic level, this mechanism allows “rewiring” existing or even creating new neural circuits [4]. Particularly, neurofeedback rebalances activity of the thalamocortical and septohippocampal systems involved in a “feedback loop” and restores stimulation-inhibition equilibrium. Neurofeedback targeting mood often relies on relatively unspecific actions, namely, reduction of stress, anxiety, and fear that implies improvement of limbic lobe abnormalities [4].

From the perspective of specifically EEG mechanisms, Markiewcz [4] proposed a simplistic classification of brainwaves as either normal (alpha, beta-1, and SMR) or pathological (delta, theta, and beta-2). The implication of this approach may be that treatments should upregulate the former and downregulate the latter. This definition is partly supported by a review by Marzbani et al. [16] that linked high beta with intensive cognitive load, hyperalertness, and anxiety while theta with creativity and insight on the one hand and depression, anxiety, and distractibility on the other hand. The idea of stimulation-inhibition control [4] in the case of EEG leads to classification of the majority of neurofeedback protocols as either ones promoting relaxation and focus (alpha/theta, alpha protocols) or ones for activation and concentration (frontal alpha asymmetry, SMR-beta, theta/beta protocols) [16]. Alpha band is of particular interest for neurofeedback for depression treatment including occipital alpha indicating calm resting state and frontal alpha showing prominent emotional and motivational asymmetry.

Infralow-frequency neurofeedback is supported by the data of resting-state fMRI studies which showed that frequencies that are typically filtered out of EEG are related to the global network activity and may be used for neurofeedback; namely, Othmer et al. [27] claim that infralow-frequency (less than 1 MHz) neurofeedback supports the restoration of the correct brain regulation that can improve most neurological and psychiatric conditions.

Marzbani et al. [16] argue for the lead-specific functional role of EEG linking emotional processes primarily to activity recorded from the frontal leads which makes them, especially the ones located in the left hemisphere, an appropriate target for a neurofeedback treatment of depression. Most studies of emotional alpha asymmetry confirm its validity only in frontal (F3/4, Fp1/2, and F7/8) and sometimes in anterior temporal lead pairs (e.g., [25, 86–89]). Othmer et al. [27], on the contrary, propose highly personalized treatment implying that different leads (primarily, parietal-temporal, temporal-prefrontal, and interhemispheric temporal bipolar ones) may be employed dependent on certain conditions and sequential switching of the electrode placements over the course and even within session must take place.

Methodical issues are still of great interest for EEG neurofeedback. Arns et al. [18] claim that, in EEG neurofeedback, quality of signal acquired is an important factor of intervention outcomes along with learning variables. Optimal number of sessions, duration of intersession breaks, strategy of reinforcement (yet some authors recommend mean + 30% [4], and some evidence prefer manual threshold adjustment over automatic), feedback presentation, and intra- and intersession progress quantification are the subjects to further research. Lack of spatial specificity is also evident in traditional neurofeedback, while spatial resolution may be increased with inverse solutions like LORETA or blind source separation.

Various EEG neurofeedback protocols have been tested as depression treatments, and a substantial body of documented studies exists. Markiewcz [4] in a recent review stated that neurofeedback is effective in many psychiatric conditions influencing psychological variables such as stress and anxiety. The author summarized some EEG (mainly alpha power-based) and fMRI neurofeedback studies of depression treatment assuming its efficacy in both affective and cognitive domains. An uncontrolled study involved a mixed sample of 77 adult psychiatric inpatients including 19 clinically depressed ones (different diagnoses, neurofeedback protocols, and course lengths were taken together), which showed reductions in depression as well as other clinical and psychological benefits [90]. However, mixing different protocols and different combinations of neurofeedback and pharmacological treatment in the same patients (69 patients were medicated and 39 received 10+ neurofeedback sessions, so some crossing took place; 48 participants underwent both *α*/*θ* and *β*/SMR training) obscured the individual effects of each in this study. So below we review studies of some EEG neurofeedback protocols allowing to estimate them separately.

### 4.1. Frontal Alpha Asymmetry Neurofeedback

Frontal alpha asymmetry in depression has been extensively tested as a neurofeedback target. The frontal alpha asymmetry protocol is the most widely used for depression treatment [5, 91, 92]. Hammond [93, 94] considered it to be promising in 2005 already. Hammond and Baehr [95] reviewed a large number of studies that mainly supported the role of frontal and parietal EEG asymmetry in emotions and depression and presented a history of asymmetry-related neurofeedback approaches. Simkin et al. [5] recommended this protocol for treating adolescent depression even though the authors hardly found any direct evidence for its efficacy in adolescents.

The idea of Rosenfeld [96] who developed the protocol was to target an EEG feature reliably traceable to the mood with respect to emotional valence. The author chose Baehr's index based on the Davidson model of frontal asymmetry as the most empirically supported one. This model deals with evidence that the right prefrontal cortex mostly processes negative emotions and controls avoidance behaviour while the left prefrontal cortex specializes in positive emotions and approaching behaviour patterns. Yet this difference is mostly evident in leads based upon the dorsolateral prefrontal cortex; the effect is believed to be related to its downstream connections, i.e., to the ventromedial prefrontal cortex, the orbital frontal cortex, the anterior cingulate, the hippocampus, and the amygdala. The second assumption supported by concurrent fMRI-EEG studies (e.g., [97]) was that beta brainwaves reflect activity of brain areas and alpha oscillations are present mostly in idle regions [93]. Imbalance of frontal cortices' activity in depression may be to some extent caused by the structural abnormalities of the left frontal pole [8] and, from a genetic perspective, by polymorphism of 5-HTTLPR coding serotonin transporter [98]. Albeit a large number of studies replicated the link between right-sided prefrontal dominance and depression, a recent meta-analysis demonstrated no such an effect [99]. Note that the failure to approve the frontal alpha asymmetry as a diagnostic biomarker of MDD does not necessarily decrease its influence as a state marker of motivation and emotions and as a trait marker of readiness to respond with certain emotions and behaviour under relevant circumstances.

Further studies of the frontal asymmetry nature showed that it is better explained by approach-withdrawal motivation and by behavioural activation/inhibition system activity than by emotional valence (e.g., [100]). However, in most cases, positive emotions are related to approach motivation and behavioural activation; thus, the link between frontal EEG asymmetry and emotion valence still remains strong, even though indirect. The capability model claims that asymmetry is present in emotionally salient context only, though understanding of this context is broad and even includes some resting state conditions [101]. Thus, the training situation, especially in depressed patients, should be assumed to be a salient enough context.

So training of positive emotions could be either increasing the left frontal beta power while decreasing the right or increasing right frontal alpha (suppressing right frontal activity) and decreasing the left. Alpha was chosen as a target for practical reasons such as being less prone to EMG contamination (this approach was criticized by Walker et al. [8] for its indirect action compared to beta upregulation in the left hemisphere). Two versions of alpha asymmetry index may be used for such asymmetry training: a normalized A1 = log *R*–log *L* and non-Gaussian A2 = (*R*–*L*)/(*R* + *L*). The relation between mood and both versions of the index has been validated in a series of studies. These scores are highly intercorrelated (*r* = 0.98), so the choice of one or another does not make much difference [96]. It is noteworthy that an index similar to A2 has been developed using a F3/F4 alpha coherence to Cz instead of alpha power. Albeit correlated with a depression severity to a greater extent than a classical A2, it has not been used in a neurofeedback treatment of depression yet [8].

Some problems were reported for the alpha asymmetry. First, it may be not evident in mixed anxiety and depression, which is a rather common case [8]. Second, early studies by Rosenfeld [96] demonstrated that both currently and formerly depressed subjects have abnormal frontal asymmetry, which may support the idea of hereditary nature of this feature and leads to two conclusions: (a) the ability of patients to modify this asymmetry is questionable because it does not change in remitted patients; (b) it is unnecessary to influence it because some people manage to improve without asymmetry changes. Another study supports a vision of frontal alpha asymmetry as a state depression marker, not a trait one (e.g., [38]). Values of left-right frontal alpha asymmetry also tend to have prominent interindividual difference which may undermine the usefulness of index as a biomarker and a target for neurofeedback. Some concerns also exist related to the determination of “normal” (target) asymmetry score for each patient. Current evidence suggests that some subsamples of depressed patients feature disrupted interhemispheric balance while others do not, and no marker allows to reliably differentiate them prior to EEG recording [29].

It is also noteworthy that neurofeedback was not a sole treatment in Rosenfeld intervention. Courses also included training of diaphragmatic breathing and autogenic training (self-suggestion of relaxation and hand warmth). Each session was divided between the neurofeedback and psychotherapy approximately at 50 : 50 ratio. During the neurofeedback session, a strategy of self-regulation was explicitly suggested, namely, “to focus on pleasant, unemotional imagery” [102]. All these additions made it hard to distinguish between effects of the neurofeedback per se and effects of other relaxation techniques, psychotherapy, and simply following the suggested strategy.

A number of proof-of-concept studies were conducted at the early stages of the protocol validation. First, the ability of healthy people to voluntarily upregulate asymmetry index was tested. In two studies with different approaches to alpha estimation [103], 9 of 13 subjects learned to achieve 0.85 standard deviations gain of the index in order to increase the monetary reward linked to their performance during four training days. In a further replication, the volunteers' ability to downregulate asymmetry and its relation to mood were also established [96]. In a study by Allen et al. [104], groups of 9 healthy women each, one upregulating and one downregulating frontal alpha asymmetry within 5 sessions, gained some control of the signal. On the 3rd and 4th sessions, groups significantly differed in alpha asymmetry, and to posttreatment, participants who activated the left hemisphere (trained more right-sided alpha) had more prominent positive emotion while viewing emotional films and more “smiling” and less “frowning” muscle activity.

Subsequent studies involved small clinical samples or were single case trials and utilized the same standardized protocol [96]: a total of 30-60 sessions (twice a week) that consisted of an initial 15- to 30-minute period of diaphragmatic breathing and thermal biofeedback, followed by a frontal alpha asymmetry trial and a psychotherapy session including discussion of the biofeedback-related experiences. The A2 was utilized, derived from the F3 and F4 leads referenced to Cz. Audial feedback occurrence condition was A2 > 0, and sound pitch got higher with higher values of A2. Some evidence was collected for a portion of time when A2 > 0 to be a reliable and valid measure of a disruption severity/improvement degree [96].

These and some ideologically similar trials provided a preliminary support to authors' assumptions, demonstrating the protocol's ability to decrease depression [105, 106] and change the approach/avoidance behaviour balance [106], as well as indicating the stability of patients' remission during the several years' posttreatment [102]. These data are not mentioned in subsequent text and tables because either no effects on depression were measured or sample sizes were too small.

In a recent randomized double-blind study [107], students succeeded in increasing relative right activity (left alpha over right), but not the left one required for the depression protocol. Moreover, the acquired training skills were not retained until follow-up. The right activity neurofeedback group also failed to reduce subjective stress response, while the left and placebo groups managed to do this, consistent with the current understanding of relations between asymmetry and mood. In another single-blind study [108], adult women randomized to the up- or downregulation group demonstrated an ability to regulate frontal alpha asymmetry in a band- and lead-specific fashion, but failed to maintain changes after the neurofeedback session and experienced no mood modification.

Mennella et al. [109] mentioned that the majority of preliminary studies of frontal alpha asymmetry protocol demonstrated participants' ability to modulate asymmetry index though it had no impact on the current mood of trainees measured with PANAS. The authors also cite some evidence that asymmetry may be caused by excessive right prefrontal activity related to anxiety and by inferior left prefrontal activity more relevant to depression. So in a course of neurofeedback, not only the asymmetry index changes but also separate left and right frontal alpha power modulations may be of clinical significance. The authors' own randomized study [109] showed that healthy females improved on mood after frontal alpha asymmetry training and not after the alpha power upregulation training.

Some researchers modified the protocol, e.g., by additions such as beta-3 band downregulation to improve anxiety symptoms [91, 98] or central beta-1 upregulation [98] to strengthen motivation and executive functions or by shifting focus to left beta upregulation [110] with additional photic stimulation [106]. Hammond [93] in a review summarized their own study based on [106] protocol and showed that unmedicated subjectively depressed adults improved on the MMPI depression score posttreatment. A few studies [91, 98, 111] utilized slow/fast brainwave balance within one lateralized lead instead of left-right balance within a frequency band. Protocol by Dias and van Deusen [91] has anecdotal support from a single case. Twenty sessions of neurofeedback aimed at increasing beta-1 power at C4 with eyes open and alpha/beta 3 ratio at P4 led to increase in alpha and beta-1 powers with simultaneous decrease in beta-2 power and improvement on depression, anxiety, and quality of sleep [98]. Below we discuss existing studies of frontal alpha asymmetry neurofeedback and few trials of other protocols based on emotional frontal EEG asymmetry concept.

#### 4.1.1. Major Depressive Disorder (MDD)

First, a pilot trial [38] enrolled 9 patients with MDD who underwent 15 to 30 sessions, 30 minutes each, of visual A1-neurofeedback, within 10 weeks. No specific self-regulation strategy was suggested. Four patients responded and four remitted after the treatment, two felt no effect and withdrew. The group had significantly reduced mean depression scores of Quick Inventory of Depressive Symptomatology, Self-Report-16 (QIDS-SR16) screening test. A1 improved between sessions and within each session, and this EEG feature correlated with QIDS-SR16, though participants' ability to control A1 within session did not change.

In another study [112], 24 MDD patients with high anxiety scores who received 10 biweekly sessions of alpha-asymmetry neurofeedback were compared with ones who underwent high-beta downregulation neurofeedback and with a control group that had no additions to a standard treatment. The experimental group showed a decrease in BDI-II scores and in anxiety estimates compared to passive controls. No difference in asymmetry score changes was evident between groups.

The core idea of the asymmetry training was also used in a protocol of Cheon et al. [113]. In their uncontrolled trial, 20 participants with MDD received a two-month course of two or three neurofeedback sessions per week. Each session included 30-minute F3 beta power upregulation followed by a half-hour Pz alpha/theta crossover training. Patients improved on BDI-II scores, Clinical Global Impression scale, and Hamilton anxiety and depression scores; however, frontal alpha asymmetry did not change significantly. According to HAM-D norms, 35% and 75% of patients responded and 15% and 55% remitted after one and two months of training, respectively.

#### 4.1.2. Depressive Disorders Other Than MDD

In a randomized study [114], 24 patients with different depressive disorders were assigned to 10 (two-weekly) half-hour sessions of either A2 audial neurofeedback (*N* = 12) or placebo “psychotherapy” (*N* = 10). The posttreatment neurofeedback group had been encouraged to self-train the mental state related to high alpha asymmetry scores for one month after completing treatment, whereas the placebo group had been receiving real psychotherapy that month. One patient in the neurofeedback group achieved full remission and four more partially remitted, while two controls also partially remitted by the end of the study. The experimental group, but not the placebo one, gained more right frontal alpha power in both eyes-closed and eyes-open conditions and improved on BDI-II, HAM-D, and Automatic Thought Questionnaire scores. Some cognitive features also improved in the neurofeedback group.

Another trial [115] employed an uncommon modification of the protocol for usage with a portable Emotiv EPOC device, yet based on the idea of asymmetry-locked mood change so we review it in this section. Ten late-life depression patients of mean age of 84 participated in the study, and six completed a course of ten two-weekly 15-minute neurofeedback sessions. Subjects tried to increase both loudness and tempo of their favourite music through augmenting alpha power asymmetry and frontal beta/alpha ratio assumed to be related to a level of arousal. Five of the six patients improved on BDI scores (insignificantly: -17% on average; though the authors argue that results of one patient who was not depressed at baseline should be eliminated and that would lead to significant pre-post difference) and demonstrated within-session increases in valence and arousal EEG measures and start-to-finish decrease of the left frontal alpha power.

Results from this section are summarized in Table 3.

### 4.2. EEG Power-Based Neurofeedback Approaches

This section reviews the studies of three submodalities of EEG neurofeedback, namely, alpha-theta neurofeedback, alpha upregulation neurofeedback, beta/sensorimotor upregulation neurofeedback, and beta suppression. Marzbani et al. [16] in their recent review state that training to augment alpha and theta powers and concurrently decrease beta power is a widely used neurofeedback protocol for treating depression. Alpha-theta protocol (rewarding greater occipital theta power, alpha power, and theta power over alpha power) gained popularity as a treatment for addictive disorders. Originating from the studies of practicing meditators, the theta-alpha crossover (theta power is greater than alpha) further served as a valuable neurofeedback target. Alpha-theta training is generally preceded by a few sessions of thermal biofeedback in order to help participants learn relaxation techniques using easier to control autonomous signals. The psychological mechanism of protocol action is usually described as providing deep relaxation, diminishing stress, and granting access to “unconscious” thoughts [116, 117]. According to the authors, the protocol drives *β*-endorphin level reduction that is related to a lower stress. Raymond et al. [118] state that anxiety reduction is a mostly nonspecific effect of alpha-theta neurofeedback, which may be even greater in sham neurofeedback, while energizing and depression decrease are more specific ones. Mood changes were reported as easy to achieve with this protocol, while personality changes require prolonged training.

Studies in substance-dependent patients [116, 117] have shown some improvements on subjective estimates of depression, and a relatively recent small-sample sham-controlled study of healthy volunteers supported the specificity of mood changes and presence of within-session improvement in alpha/theta ratio [118]. However, the only trial to implement this biomarker in clinical depression treatment was performed in 2005 [119].

Training on a single alpha band is also generally understood as a relaxation-providing one. The absence of a theta element means that no intention to induce trance or drowsy state is implied, and the patient stays fully conscious. The choice of upper alpha band only for self-regulation may be motivated by a need to improve alertness, activity levels, and cognitive performance (an “average” of alpha- and beta-upregulation training effects).

Some evidence against the protocol are the results of Bhat [120] who found that alpha power upregulation neurofeedback leads to worse results in anxiety diminishing compared to anxiolytic medication (50 patients were assigned to each group). Commenting on this result, the author pointed to the fact that patients were predominantly diagnosed with mixed anxiety and depressive disorder and claimed that there is no evidence for the effectiveness of such a protocol in treating depression symptoms.

Results in subclinical depression are mixed, two randomized controlled studies showing no improvement in depression estimates [109, 121]. One uncontrolled trial that involved a sample of female Canadian aboriginals who underwent intense training of closed-eyes alpha in central and occipital leads evidenced decreased estimates of subjective depressed mood [122]. One more randomized controlled study of alpha power upregulation at peak frequency in students with elevated BDI-II score though no formal diagnosis of depression showed improvement on BDI-II, rumination, and executive functions in the training group and not in the no treatment group [123]. Only one study in clinical depression was found that featured a cognitive outcome measure, as opposed to emotional [124].

Such symptoms of depression as lack of energy, workability, and cognitive deficiencies require a more activating EEG protocol. Lack of motivation and executive functions in depression may be linked to deficient beta EEG oscillations [91]. This idea is supported by the fact that 20 Hz audial-visual stimulation leads to improvement in seasonal affective disorder in both emotional and social domains [125]. SMR was poetically defined as related to “quiet body and active mind,” and this activity is also deficient in mood disorders [126]. Cantor and Stevens [127] in a crossover study demonstrated that 20 sessions of audial-visual stimulation at 14 Hz reduce BDI-II scores in adult participants initially high on depression and free of psychotropic medication, so these results support the idea of SMR upregulation for depression treatment.

Grin-Yatsenko et al. [128] cite papers by Othmer et al. inspired by Sterman SMR training seizure protocol on the one hand and Ayers' for brain traumatic injury with beta-1 upregulation on the other hand. The final edition of the protocol involved 15-18 Hz for the left prefrontal area (Fp1-C3) and 12-15 Hz for the right parietal cortex (C4-Pz). In two studies by Othmer (cited and successfully replicated by Walker et al. [8]) that were the first ones in the field of depression self-regulation treatment, neurofeedback aimed at decreasing theta and increasing beta-2 (15-18 Hz) at C3 was effective in treating depression in a population of patients with different affective disorders. Alpha (8-11 Hz) inhibition was found to promote more activation in trainees, and beta-2 upregulation on site T3 with SMR upregulation at T4 demonstrated good results in bipolar depression. Side effects of inappropriate training were also discussed, such as anxiety, aggression, and somatic symptoms for beta overtraining and depression and irritability for excessive SMR training.

Aside from one study of MDD patients discussed below, three relatively relevant trials were performed. First, in an uncontrolled study, 183 patients aged 12 to 70 and not responding to antidepressant treatment strikingly benefitted from a training involving closed-eyes beta power in the 15-18 Hz range upregulation with concurrent theta power downregulation at Fp2 (assumed to target anterior cingulate). By the posttreatment point, 84% patients remitted; however, it is noteworthy that both initial condition and remission estimation were based on the results of Depression Self-Rated Test [129]. Second, in a well-designed study, fibromyalgia patients who received SMR training improved on HAM-D and BDI greater and sooner than the escitalopram group and demonstrated a corresponding EEG change [126]. However, healthy volunteers failed to decrease BDI-II scores after the SMR neurofeedback training unless it was combined with transcranial direct current stimulation [130].

Beta power suppression is a rather young neurofeedback approach. The rationale for its usage is the link of high-beta distributed broadly over the cortex with an anxiety and, possibly, some cognitive features of depression, like rumination [112].

Below we discuss the existing studies in clinical depression based on regulation of the certain EEG frequency bands.

#### 4.2.1. Major Depressive Disorder (MDD)

First, in a controlled study, Hashemian and Sadjadi [119] trained 28 adolescents diagnosed with major depression to decrease EEG alpha/theta ratio at F3 with audial-visual feedback without biofeedback pretraining, so the protocol was rather different from Peniston's. Each participant underwent 20 half-hour sessions; subjects were randomly allocated to the real or placebo (recordings of feedback from other people) groups in addition to fluoxetine treatment, *N* = 14 each. Both groups improved on HAM-D, and real neurofeedback was not significantly superior to sham.

In a controlled study without a randomization, a course of eight 32-minute occipitoparietal upper-alpha upregulation sessions helped 40 MDD patients to improve speed and performance in a working memory task compared to the nonintervention group (20 subjects with MDD). The experimental group also increased alpha-band power and current density in alpha band in the subgenual anterior cingulate. Alpha power increases were evident both within individual sessions and from session to session. Surprisingly, behavioural improvements correlated with beta rhythm power enhancement [124].

A protocol combining the traits of a few others, namely, Scott-Kaiser's modification which is alpha-theta pretreated with SMR and lateralized frontal brain activity training originating from the emotional frontal asymmetry concept, was utilized in a study by Lee et al. [45]. Twelve patients with a treatment-resistant MDD received 12 to 24 one-hour sessions of combined game-based SMR or beta neurofeedback and classical alpha-theta in addition to treatment as usual. On weeks 4 and 12 of treatment, neurofeedback participants showed lower HAM-D and mental problem severity scores and higher emotional quality of life than controls who received placebo supportive psychotherapy and treatment as usual. The neurofeedback group was significantly higher on BDI-II on baseline but did not differ from the control group at week 1 and subsequent time points. As much as 58% of neurofeedback group participants responded (50% reduction of HAM-D) and 50% remitted (HAM-D <7) by week 12, while only one patient in the control group did (the difference is significant for response rate). During the treatment, neurofeedback patients had slightly decreased serum levels of brain-derived neurotrophic factor (BDNF) and the control group had slightly increased levels resulting in no within-group effect but significantly different change of BDNF serum concentration. The authors in fact do not interpret this finding and discuss only an absence of significant within-group changes.

In a Wang et al. [112] study, a P3-P4 high beta (20-32 Hz) inhibition protocol was tested. 23 anxious MDD patients who underwent 10 sessions of neurofeedback improved on BDI-II and Beck Anxiety Inventory estimates, though so did patients of the frontal alpha asymmetry group. Both groups outperformed the control (no neurofeedback) group on psychological metrics. The beta neurofeedback group managed to decrease the power in the target band at P3 site. Reanalysis [131] of the data from this trial demonstrated that training effects persist to some degree resulting in decreasing posttraining high-beta powers within the course and are limited to beta band (both low and high). A decrease in high-beta was also shown to be correlated with improvement in BDI-II total and cognitive depression estimates.

Results from this section are summarized in Table 4.

### 4.3. Other EEG-Based Neurofeedback Approaches

Below we discuss some other rather variable EEG neurofeedback protocols. Interestingly, Walker et al. [8] summarized the quantitative EEG (qEEG) data on various subtypes of depression and recommended certain protocols for each one; namely, 2-7 Hz downregulation and 15-18 Hz upregulation at F8 site for endogenous depression. “Cognitive” depression was linked to the same bands in a contralateral site (F7). For bipolar affective disorders, separate markers were named for depression (slow activity at F3) and for mania (excessive frontal beta) which were expected to be compensated for in a neurofeedback course. Comorbid anxiety was assumed to be reduced with beta downregulation training with no respect to lead. The well-known Rosenfeld frontal alpha asymmetry protocol was recommended for reactive depressive states only. It is worth noting that this marker is the only one treated by the authors as a state one. This list does not exhaust the options of EEG-based neurofeedback for depression treatment, including the slow cortical potential- (SCP-) and quantitative EEG-associated ones.

SCP neurofeedback training is a broad term covering a primarily seizure-countering protocol that had some positive side effects on depression estimates. Three sessions of SCP training were demonstrated to be efficient in regulating negativity amplitudes and hemisphere specific (F3/F4) in 16 healthy people with no regard to the presence or absence of suggested emotional strategies. Those who were higher on a withdrawal scale also had greater right hemisphere negativity [132]. On average, less than half of the healthy participants learned to produce a voluntary differentiation of SCP amplitudes in C3 and C4 during a short-term course. Twenty-one of 45 were successful in 80 trials of continuous feedback and 20/48 managed self-regulation in 120 trials of intermittent feedback. Differentiation was shown to be greater in immediate continuous feedback condition [133]. SCP training for epilepsy may lead to some improvement though two controlled studies failed to demonstrate its specificity [35, 37]. One preliminary study of SCP neurofeedback in MDD is reviewed further [42]. Bostanov et al. [134] recently showed that mindfulness meditation leads to changes in late contingent negative variation (CNV) potential amplitudes, and these differences are strongly correlated with a long-term improvement on depression symptoms, so CNV neurofeedback may also be of use in depression.

Grin-Yatsenko et al. [128] presented a pilot study of SCP neurofeedback training targeting infralow frequencies (below 0.1 Hz) which were hypothesized to be related to fMRI resting state network activity, primarily the default-mode network (DMN). In three unmedicated patients, 20 sessions of training decreased the clinical estimates of depression (MADRS and HAM-D) and excessive frontal and central theta and alpha powers. In a group of drug addicts sentenced for robbery, some specific improvement on HAM-D score was shown after infralow-frequency (0.01-0.02 MHz) neurofeedback with a concurrent suppression of a number of bands in the 1–40 Hz range [135]; however, this study remains the only one in this submodality dealing with depression estimates.

LENS is a technology that promotes a short-term electromagnetic feedback estimated as 10^–7^ of TMS intensity delivered via the same lead used for EEG recording which is subliminal for participants' perception [136]. LENS is thought to suppress EEG activity in the sites of its greatest prominence which hypothetically improves cortical control over the subcortical areas, which is consistent with the current understanding of depression neural mechanisms [137]. The nature of the LENS (stimulation or neurofeedback modality) and its effectiveness are questionable. An article [136] on an uncontrolled group trial with treatment-resistant depression patients is a substandard one for a scientific publication in failing to report the sample size and the baseline characteristics as well as any formal improvement criterion. An uncontrolled study showed an improvement on mood disturbances score (index including depression) in a mixed group of 100 patients with different conditions after a course of LENS [137], and we have found no other relevant trials published to date.

A few studies focused on the qEEG neurofeedback in depression. Two uncontrolled trials showed an improvement on depression estimates in adult attention deficit disorder (ADD) or attention deficit and hyperactivity disorder (ADHD) [138] and postconcussion syndrome [139]. Some evidence for qEEG self-regulation in MDD is reviewed below.

#### 4.3.1. Major Depressive Disorder (MDD)

A proof-of-concept study [42] showed that 8 medicated inpatients with MDD or bipolar depression were able to control the SCP negativity/positivity within 20 game neurofeedback sessions. Each session comprised a total of 110 of 8-second feedback and transfer trials with a few seconds gap between the trials. No cognitive strategy was suggested. Participants received small financial compensation linked to the self-regulation performance. Depressed participants acquired control over the SCP within the first five sessions and then nonsignificantly strengthened it, while the healthy subjects failed to regulate the SCP within five sessions. Although there is some evidence of a deficit of slow event-related potential (ERP) amplitudes in depression, the clinical value of this protocol remains unclear.

Paquette et al. [140] in their uncontrolled study applied an idea of qEEG in an unusual way related to group abnormalities rather than individual ones. First, the authors compared data of their MDD group (*N* = 27) to ones from the normative database, estimated group differences, and localized their sources with LORETA. These abnormalities were excessive 18-30 Hz beta in the frontal areas originating from the middle frontal, orbital frontal, insular, anterior cingulate, and some cortical temporal areas; the hippocampus and amygdala were targeted in training of downregulation of 18-30 Hz band in AF3, AF4, T3, and T4 leads in eyes-open condition (this also partly matches Othmer's [27] choice of leads and bands for downregulation). The course comprised 20 sessions each containing 8-10 blocks 3 to 4 minutes each. The first 10 participants were recommended to relax with some breathing exercises, and the last 10 to use feedback in an attempt to decrease the intensity of the negative thoughts and feelings. Patients were concurrently medicated. At posttreatment, 74% of patients remitted on DSM-IV criteria, and the group managed to significantly decrease average BDI-II estimates by 72.9%. EEG beta power diminished significantly after training and LORETA *z* scores for all predefined sources fell into normal range (<2) for responders only, while nonresponders increased high beta in the left frontal areas. Right frontal and temporal pole beta activity reduction correlated with the improvement on BDI-II scores.

Results from this section are summarized in Table 5.

### 4.4. Conclusion

The frontal alpha asymmetry training, despite being the most popular in the field and based on a relatively well-established biomarker, in fact lacks strong evidence of effect in depression. The majority of the relevant studies were either single cases, or involved insufficient samples, or uncontrolled. Both controlled studies [112, 114] demonstrated some improvement on subjective measures of depression and one [114] also documented some hypothesized EEG dynamics. However, the superiority of the neurofeedback group in depression improvement without between-group difference in frontal alpha asymmetry changes in another study [112] suggests the importance of the exercise itself, not of achieving a certain EEG modification, and may be evidence against the protocol action specificity. Standard course lengths lied within a range of 10-30 sessions, which suits the opinion of EEG neurofeedback as a hard-to-learn modality. The remission rates in MDD were nearly 50%, which seems to be an impressive result, though the studies' reported remission rates were uncontrolled [38, 113]. The existing studies in major depressive disorder and in other depressive disorders fit level 2 of evidence (“possibly efficacious”). Nonclassical protocols based on the idea of prefrontal emotional asymmetry may warrant attention in the future though currently they fit evidence level 1 or 2.

No specific neurofeedback-related effects on depression were evident for other EEG protocols as well. Few studies showed EEG neurofeedback superiority over no treatment condition [112, 124], and SMR+alpha-theta protocol was superior to a placebo psychotherapy [45], while the effect of alpha-theta alone was comparable to one of a sham neurofeedback [119]. Two studies indicated the expected EEG changes [112, 124, 131], and high-beta suppression protocol even demonstrated a correlation between the EEG and depression dynamics [131]. Nevertheless, the only study within the alpha-theta submodality that targeted depression as a primary complication (adolescent MDD) fits level 2 (“possibly efficacious”). For the alpha upregulation protocol in a major depression, the evidence level is also 2 (“possibly efficacious”) referring to only one study. Note that only cognitive improvement was assessed in that trial, not emotional. SMR upregulation combined with the alpha-theta was tested on MDD patients in a single controlled study that also matches level 2 of evidence (“possibly efficacious”). The same is true for the high-beta downregulation protocol (level 2, “possibly efficacious”). Some studies on other EEG submodalities also exist, while only qEEG approach was tested in MDD population (level 2, “possibly efficacious”). Note that the restriction of the estimates to level 2 is more related to quantity of the studies than to their quality, given that each submodality reviewed was tested in clinical depression in a single trial. Other submodalities still do not have empirical support; namely, the SCP neurofeedback is yet to be studied in clinically depressed samples. The value and the mechanism of action of ILF and LENS treatment require subsequent validation prior to bringing them to the populations of depressed patients.

## 5. Real-Time fMRI Neurofeedback (rt-fMRI NFB)

A novel neurofeedback modality has been developed in the last two decades, allowing to access the metabolic signals from the local brain areas via fMRI [141] (or analogous methods such as a functional near-infrared spectroscopy, fNIRS) to treat some conditions, including depression. The fMRI neurofeedback learning, in contrast to EEG, was demonstrated to be possible within a single session, which may be due to a baroreceptor system impact on blood flow regulation. Some evidence from animal studies supports this hypothesis [23]. Approximately 50-75% of human trainees manage to change the activity of the target brain area by means of the rt-fMRI [142]. In most rt-fMRI studies, participants' ability to regulate certain brain regions [23] and the presence of the blood oxygenation level-dependent signal (BOLD) regulation learning in the course of the rt-fMRI NFB and its preservation to transfer sessions [7] has been demonstrated, though the data on the corresponding mood changes (PANAS) and changes in a perceived stimuli valence were inconsistent [7].

Some methodological issues critical for the development of the rt-fMRI neurofeedback as a clinical tool exist, namely, proving a reliable rt-fMRI signal and progress in self-regulation skills in patients, testing learning success appropriately, demonstrating positive clinical outcomes, implementing high-quality designs, and sharing resources and elaborating the common standards [143]. Obviously, more studies involving clinical samples are needed because currently 64% of trials recruit healthy volunteers only [142]. Linhartová et al. [3] claim that the success of the fMRI neurofeedback learning for emotional regulation depends primarily on the target RoI, emotional regulation task, and population undergoing the training. According to Arns et al. [18], optimal regions of interest, course length, trial design (including an appropriate placebo condition), the role of instructions, and feedback presentation should be researched thoroughly. Instead of trying to cover all these issues, we will discuss in detail two of them that are particularly important for the rt-fMRI neurofeedback treatment of depression, namely, the choice of a region of interest and a cognitive strategy suggestion.

The emotional domain is the most researched one with the rt-fMRI neurofeedback [142], which is especially true for a positive emotion induction task [3]. Yet Linden [29] claimed that neither fMRI nor EEG established a reliable biomarker to target depression symptoms; still, a set of “classical” emotional areas does exist, and choosing one of them may be a “thumb rule.” This list includes the amygdala, the anterior cingulate, the anterior insula, and the orbitofrontal cortex. The most popular RoI for depression treatment is the amygdala. Amygdala upregulation was found to be valid in terms of education, maintaining the skill in transfer sessions, and association with emotional variables [3]. A recent review by Barreiros et al. [144] shows some evidence of trainees' ability to regulate their emotional responses by means of amygdala-targeted rt-fMRI neurofeedback, though studies in this field were found to be rather heterogeneous. A good example may be a study by Lorenzetti et al. [145] who used an uncommon approach to feedback (colour change of the virtual environment) and successfully trained volunteers to selectively activate the amygdala and the septohippocampal area and to reproduce the neural patterns related to complicated emotions, namely, tenderness and anguish. According to a review by Linhartová et al. [3], emotional regulation in healthy participants is frequently being performed via the insular cortex. Anterior insula upregulation unspecific to emotional valence was found to be valid in healthy participants. Some evidence was also collected for anterior cingulate and orbital frontal cortex regulation success. Prefrontal cortex training generally influences other emotional regions, e.g., the amygdala, not a prefrontal cortex activity itself (that is also expected to occur in frontal alpha asymmetry EEG neurofeedback). A number of the amygdala rt-fMRI neurofeedback studies [146–151] are reviewed below along with a few ones targeting the prefrontal cortex [152, 153], insula [36, 152, 154], and anterior cingulate [151, 154].

Aside of the well-known RoIs, depression is believed to be related to disruptions of the frontoparietal network comprising the lateral prefrontal cortex, anterior cingulate, and intraparietal sulcus area, which are assumed to be a neural substrate for a working memory, goal-directed actions, and performance monitoring. These abnormalities may be related to variations in monoamine oxidase and D2 dopamine receptor [5]. From the neuroimaging perspective, targeting certain brain regions or their interconnections may counter depression symptoms.

In a review by Linden [29], a number of targets likely suitable for the real-time fMRI biofeedback for depression treatment were named. Among them were the lateral prefrontal cortex, the ventral striatum, the subgenual anterior cingulate, and the amygdala as parts of cognitive control, reward, or limbic circuitries, respectively. The most popular model of emotional regulation used in depression treatment is the frontolimbic circuit comprising higher emotional control centres, namely, different prefrontal areas and the anterior cingulate—and limbic structures, primarily the amygdala. The amygdala is spontaneously driven by emotional stimuli, while top-down influences from frontal cortices may reduce or increase its activation [148, 149, 155, 156]. The activity levels in the amygdala and the anterior insula most probably indicate the emotion intensity with little regard to its valence, while the lateral prefrontal activity points to a prominence of emotional regulation efforts [3]. Evidence for this pathway's relevance to emotional disturbances was that ventrolateral prefrontal cortex-amygdala connectivity neurofeedback was successful in decreasing the levels of subclinical anxiety [157].

Sacchet and Gotlib [13] in their review point to possibilities of utilizing the functional connectivity-based neurofeedback to improve the known abnormalities in networks' hyper- or hyposynchrony in depressed subjects. For these purposes, more “rapid” MEG and EEG and hybrid technologies such as fMRI-EEG also may be of use. A trial of resting state functional connectivity normalization between the left dorsolateral prefrontal cortex and the left precuneus/posterior cingulate area was published [158] suggesting an improvement in target signal control and nonsignificant decrease of depression level, which could be an artifact of small groups. One controlled study demonstrated a trend to successful reduction of functional connectivity between the precuneus and the temporal-parietal junction in healthy participants. Neural effect was paralleled by improvement in rumination, though prominence of these effects was not correlated [159]. Koush et al. [160] published a promising pilot trial on regulation of effective connectivity (which is functional connectivity with respect to a direction of link between RoIs) within the prefrontal-amygdala system (the frontolimbic circuit).

Sacchet and Gotlib [13] paid some attention to machine learning procedures used to differentiate emotional states from one another and produce a feedback signal based on this classification. Feedback collection from the volumes with complex and dynamically changing shape, e.g., results of the multivoxel pattern analysis (MVPA), was mentioned as one of the future directions of the fMRI neurofeedback [29], though, to our knowledge, still no publications of treating depression with MVPA neurofeedback are available. Schnyer et al. [161] examined the MVPA-based neurofeedback for correction of negative attentional bias in healthy participants scored high on BDI-II. Their task was to reproduce the neural pattern of ignoring the sad faces. Unfortunately, no statistics on depression estimate improvement were included in the article. Machine learning techniques, such as the linear support vector machines, were mentioned by Arns et al. [18] as a way to produce the fine-grained regions of interest, yet also have not been tried empirically in depression. In a recently emerged field of decoded neurofeedback (feedback is given on the similarity of the current activation pattern to the desirable, e.g., “healthy” one), only one study related to emotions and none devoted to mood have been found; namely, it has been demonstrated that learning participants to adopt different cingulate activity patterns leads to correspondent changes in their preferences of facial images [162].

Linden [29] proposed that neurofeedback targets may be not curing abnormalities, but influencing the normal processes involved in disorder development (e.g., enhancing the emotion regulation or suppressing the self-other comparisons in depression). Improving the compensatory mechanisms via neurofeedback is also assumed to be a valuable strategy along with symptom countering [141, 143]. A recent methodological article [163] that developed the ideas of Linden [29] introduced a process-based framework for neurofeedback which means that neurofeedback targets not a depression severity, but a certain impaired process (anhedonia, decreased mood, lack of approach motivation, etc.) without linking it to a formal diagnosis. All compounds of the neurofeedback should be chosen according to the target process, namely, the neural target, interface, and outcome measures. This approach would simplify studying the psychophysiological effects related to a neurofeedback, especially in polysymptomatic disorders such as depression; however, it may take a long time to overcome the traditional diagnosis-centred approach.

Neurofeedback may also be integrated with other comparatively noninvasive neurotherapy tools. For instance, some evidence suggests that repeated transcranial magnetic stimulation (rTMS) varies in effectiveness based on the current cortical activation level. Interactive rTMS protocols overcome this by using special tasks to achieve a required patient's state. For MDD, emotional tasks are utilized to impact the activation of the dorsolateral prefrontal cortex, a typical rTMS target in treating affective disorders [164]. This technology may implement neurofeedback for a pretreatment. Some stimulation technologies in turn may improve neurofeedback efficiency, e.g., transcranial direct current stimulation added to SMR training leads to a decrease of subclinical depression scores [130].

One more future direction is a multimodal integration. Zotev et al. [165] presented a proof-of-concept study of a hybrid neurofeedback, based on simultaneous regulation of high beta (21-30 Hz) band F3-F4 power asymmetry (correlated with avoidance of angry faces and depressive symptoms) and upregulating blood oxygenation level-dependent (BOLD) response of the left amygdala by means of self-induction of positive autobiographical memories. Six healthy participants underwent a single training session and in general managed to regulate both signals though a threshold of significance was met only in a few runs. A recent study by Zotev et al. [151] involving two EEG and two fMRI signals concurrently is discussed in details below.

EEG neurofeedback gained some benefits from fMRI research and concurrent fMRI-EEG recording. For instance, Othmer et al.'s [27] infralow-frequency neurofeedback was inspired by the resting-state fMRI studies. In addition, a novel approach was developed to predict the fMRI activity of the amygdala from EEG recordings that allows for performing neurofeedback on a simulated BOLD signal without physically entering an MR scanner in fact, with a relatively simple EEG device, namely, “amygdala electric fingerprint” [166]. Its correlation to the real BOLD signal of the right amygdala was established, and neural and behavioural validity of the model was proved in three sham-controlled studies. Recently, Keynan et al. [167] have presented a study validating the algorithm on a large sample of healthy participants of a military training program. It is noteworthy that such an EEG modulation of amygdala response to stressful stimuli led to an increased connectivity of the amygdala to the ventromedial prefrontal cortex.

The current fMRI neurofeedback most frequently utilizes explicit instructions [142]. Yet data on a strategy role are currently insufficient; instructions also may be needed to prevent possible harmful strategies, especially in the case of amygdala or insula neurofeedback [3]. All in all, training success depends heavily on the suggested cognitive strategy for modulation of emotional regions and certain targeted symptoms. The bias towards negative emotional stimuli is well known in depression, and its correction may be a cognitive mechanism of depression treatment. On the neural level, it is reflected to the greatest degree in amygdala reactivity [148, 149]. According to Mennen et al. [168], many of the rt-fMRI neurofeedback approaches to depression influence attentional bias to some degree. One study aimed at shaping the attentional bias is reviewed below [161].

The most frequently suggested strategy in emotional rt-fMRI neurofeedback is imagery, especially autobiographical [3]. Combination of a neurofeedback and mental imagery may enhance a psychotherapeutic technique with a noninvasive neuromodulation in affective and anxiety disorders [169]. It is worth noting that mental imagery is greatly dependent on perceptual (primarily, visual) system and features the neural representation and mechanisms similar to those of perception of weak external stimuli. Thus, sensory characteristics of an image have more importance than its semantics. Imagination also partly shares the underlying neural networks with autobiographical memory and cognitive analysis of the current situation (frontoparietal).

The rt-fMRI neurofeedback protocol described by Zotev et al. [30, 151] and Young et al. [147–150] incorporates strategy of happy autobiographical memories as a means to control amygdala activation. An autobiographic memory retrieval condition is known to be related to activation and functional coupling of the amygdala, the hippocampus, and the right inferior frontal gyrus [170]. Noteworthily, Köhler et al. [171] discuss recalling positive memories and enriching them with details as a valuable psychological approach to deal with autobiographical memory disturbances in depression. To some extent, this strategy may be treated as a part of more global, namely, a supervised mental imagery that may enhance the participants' cognitive flexibility and ability to produce positive images [36].

In a randomized study by Zotev et al. [30] which was a foundation of subsequent research by Young et al. [147–150], 14 healthy participants demonstrated ability to upregulate the left amygdala with happy autobiographical memories within one session, while 14 controls who received feedback from the intraparietal sulcus did not. Further effective connectivity analysis [172] revealed increased connections of the left anterior cingulate to the left amygdala and some frontal regions of interest after the training session in the experimental group. Zotev et al. [30] findings were replicated in a study by Hellrung et al. [173] which demonstrated self-regulation learning in feedback groups and not in no-feedback group with greater success in an intermittent feedback group compared to a continuous feedback group.

Cognitive techniques of perceiving subjectively negative stimuli as positive or neutral (reappraisal, reality check, and mindfulness meditation) are found to be useful in emotional regulation [155]. A recent study [156] points to the fact that the activation of the prefrontal cortex and the amygdala as well as their functional connectivity may be modified by the cognitive reappraisal, a well-known cognitive strategy to cope with negative emotions, while for attention deployment, another studied strategy, no such link exists, and inferior parietal lobule is being activated instead. Cognitive reappraisal with a concurrent downregulation of the right amygdala activity was successfully tested on 6 healthy subjects by Brühl et al. [174] and then in a single-blind controlled study by Herwig et al. [155], though regulation success was not maintained in a transfer task and no posttraining mood change was demonstrated.

In one more study, the lateral prefrontal cortex neurofeedback with a suggested strategy of cognitive reappraisal of observed aversive stimuli produced a decrease in amygdala activation [175]. Birbaumer et al. [22] cited some studies which demonstrated that the real-time fMRI neurofeedback leads to an altered emotional perception of aversive stimuli while applied to the anterior insula and to awareness of previously subliminal emotional facial stimuli while targeting the frontoparietal brain network. However, to our knowledge, neurofeedback courses implementing the reappraisal strategy still have not been tested in depressed populations.

Ochsner et al. [176] combined the data on emotion regulation processes in a model of cognitive control of emotion. From the psychological point of view, cognitive control may influence the processes of perception, attention, appraisal, and response to emotional stimuli. “Automatic” appraisal system generating emotional responses comprises the ventral striatum, the amygdala, and the insula. Key cognitive control regions are the dorsomedial, dorsolateral, ventrolateral, posterior prefrontal, dorsal anterior cingulate, and inferior parietal cortices. Two neural mechanisms of emotional reappraisal are assumed; namely, (1) the left dorsomedial prefrontal cortex positively influences the ventromedial prefrontal cortex, and both negatively influence the left amygdala; (2) the left ventrolateral prefrontal cortex positively influences the ventromedial prefrontal cortex, and both negatively influence the amygdalae. These pathways may be mediated by striatum activity, hypothetically, in cases when the participant may get ready to aversive stimuli onset.

Last, an ability to train the amygdala up- or downregulation in the absence of explicitly suggested strategies was supported by a study wherein 35 healthy adults underwent three 40-minute training sessions which employed both continuous and intermittent feedback presentation [177]. Participants demonstrated significantly distinct amygdala activity levels in up- and downregulation blocks within a transfer session. However, direct amygdala downregulation in a study [178] of healthy women without a clear strategy yet with information that emotions are involved showed that the amygdala neurofeedback was not superior to the thalamus neurofeedback in decreasing amygdala response to aversive images, though specifically elicited amygdala-ventromedial prefrontal cortex connectivity [179].

The following sections and the final table summarize the current results on the rt-fMRI neurofeedback approaches to depression without splitting data across different protocols. Nevertheless, the level of evidence in the table and in conclusion is estimated with certain protocols taken into account.

### 5.1. Major Depressive Disorder (MDD)

Studies by Johnston et al. [180, 181] that demonstrated a possibility of regulation of emotional RoIs within the frontal cortex inspired the first clinical study in the field [152]. Eight patients with a recurrent major depression underwent four real-time fMRI neurofeedback sessions separated by 1- to 2-week gap; each session comprised four runs. The targets were the individually localized areas (top 1 : 3 activated voxels) selectively responding to positive stimuli, namely, the ventrolateral prefrontal cortex, the insular cortex, or the right dorsolateral prefrontal cortex. The initial strategy was to imagine positive scenes similar to ones used in a localizer; then, most participants moved to positive autobiographical memories and some of them to images of future success. For trainees with negative regulation success during the first run, the RoI was changed to another one positively correlated with regulation block design for the next run. This is an uncommon practice for neurofeedback studies because, instead of demanding the subjects to master the skill of activation of the required area, the area is adjusted in order to find one that the patient is able to regulate. In total, RoI was adjusted after nearly half of the first runs within session and in 1/8 cases after the second run. Four controls practiced a strategy of positive imagery without neurofeedback and outside the MR scanner. Experimental participants managed to enhance the activity in the RoI within session and not between sessions. To prove that this result is not fully attributable to the RoI adjustments, the authors also demonstrated a significant linear increase of the RoI activation within the first run. The neurofeedback group decreased HAM-D depression (Cohen's *d* = 1.5) to a greater extent compared to control participants; moreover, three trainees remitted on HAM-D scores and three more responded to treatment. Some effect, though it did not last after covariate addition, was also observed for within-session mood changes (PoMS) regardless of group [152].

One more neurofeedback study [153] targeted the left dorsolateral prefrontal area that is consonant with the frontal alpha upregulation idea and principal idea of the Johnston protocol to improve positive emotionality via cortical upregulation. A certain target was a set of voxels active during a localizer task involving executive functions and negatively correlated with the posterior cingulate activity at rest within anatomical borders of the left dorsolateral prefrontal area. Six patients underwent five sessions of neurofeedback on five consequent days. Sessions consisted of 1-3 runs, depending on the patients' ability, each run lasted 6.5 minutes. Participants improved on HAM-D and BDI-II estimates and also on rumination severity. Changes in the left dorsolateral prefrontal activity at baseline and posttreatment significantly correlated with the corresponding changes in rumination (*r* = –0.97). Our own unpublished data also suggest that some control over the left prefrontal cortex may be achieved by depressed patients during a course of 4-8 weekly neurofeedback sessions, though clinical changes are mostly inferior to those gained by participants who underwent a course of cognitive behavioural therapy.

Another important study involved rt-fMRI neurofeedback targeting the left amygdala in 14 participants (7 controls received sham biofeedback from the intraparietal sulcus; group assignment was nonrandom). The suggested strategy to upregulate the left amygdala by producing happy autobiographical memories was based on findings of Johnston et al. [180] and Linden et al. [152], but the one used was the exact same protocol as in [30]. The single training session included a practice run in the beginning, three runs in the middle and a transfer run without feedback at the end. Each run comprised alternating blocks of generating memories, neutral task (counting), and rest. Experimental subjects, compared to controls, achieved more left amygdala BOLD signal in the 2nd and 3rd training runs and the transfer run and reduced PoMS depression scores and Visual Analog Scale (VAS) sadness scores and increased VAS happiness scores pre-to-post scan. Left amygdala fMRI signal was inversely correlated with the duration of the current depression episode and TAS-20 scale Difficulty Describing Feelings. Last, the experimental group developed specific training-related activations in the left superior temporal gyrus, the temporal pole, and the right thalamus [147].

The experience of that neurofeedback protocol also changed the connectivity between the emotional areas. Initially, patients showed a hypoconnectivity of the left amygdala to a number of regions. Controls significantly improved connections of the left amygdala to the left cuneus, the left precuneus, the left pregenual cingulate, and the middle frontal gyri bilaterally. Experimental patients augmented the left amygdala connectivity to the left angular gyrus and the left precuneus. Compared to controls, real neurofeedback trainees achieved more connectivity between the left amygdala, the right parahippocampal gyrus, the right superior temporal gyrus, and the bilateral middle frontal gyrus, between the pregenual anterior cingulate and the left superior temporal cortex, the left superior frontal gyrus, and the right superior temporal gyrus. In experimental participants, the left amygdala-left cuneus connectivity was related to decreases in HAM-D score. This connectivity continued to increase after the training, and its measure was significantly positively correlated with the length of delay between training day and posttraining measurement. Moreover, in the experimental group, only increases in amygdala connectivity were correlated with decreases in depression [182]. Evidence exists that the fMRI-neurofeedback-driven plastic changes may be nonspecific in initial phases of treatment [182], but become more specific with several subsequent sessions.

The study by Young et al. [147] was continued. The frontal upper alpha EEG spectral power asymmetry known to be a marker of depression was tested for relation to depression scores and to the amygdala BOLD response asymmetry in a sample of 13 patients (experimental group of amygdala training). The difference between happy memories and rest condition EEG asymmetry in F3-F4 and F7-F8 was correlated positively with HAM-D and SHAPS anhedonia scores for dampened baseline in serious depression. Correlations between the BOLD amygdala asymmetry and EEG high alpha power asymmetry were also evident (*r* = 0.61 for F3-F4 and *r* = 0.64 for F7-F8). This finding showed that the amygdala lateralized BOLD response and EEG frontal alpha asymmetry; two well-known neural correlates of mood are interconnected and, to some degree, validate each other. Average left amygdala activity correlated with changes of VAS happiness and, inversely, of PoMS tension. Amygdala asymmetry index significantly correlated with TAS-20 alexithymia and marginally significantly with HAM-D scores. Furthermore, the EEG-BOLD correlations for the amygdala and other emotional regions (insula, orbital frontal cortex, superior frontal gyrus, and cingulate gyrus) became greater during the training session [43].

In a double-blind placebo controlled study, 36 adults with MDD were divided into experimental (amygdala feedback) and control (intraparietal sulcus feedback) groups. Eighteen experimental and 16 control participants finished the course, and one more control was excluded due to head movement. Patients in each group completed two approximately 50-minute sessions; each of them consisted of eight 8 m and 40 s runs, though only three of them were training ones. The sequence was as follows: one rest run, one baseline run, one practice run, three training runs, a transfer run, and rest run. Training runs comprised alternating 40 s blocks of rest, regular, and backward counting conditions. Experimental trainees gained a more prominent increase in amygdala BOLD response compared to controls and had higher rates of clinical remission and improvement on MADRS, HAM-D, and BDI-II assessments and Snaith-Hamilton Pleasure Scale. MADRS scores correlated with amygdala activity and with autobiographical memory performance. Patients also became able to increase the specificity of positive autobiographical memories (to remind certain place- and time-locked events) [150]. The authors mentioned a potential blurring effect of the basal vein of Rosenthal blood flow on the fMRI amygdala activity as a complication of the amygdala-targeted neurofeedback, though it was minimized by the imaging parameter adjustment [149].

Additional results included increased amygdala activity to subliminally presented happy faces and decreased to sad faces after two neurofeedback sessions in the experimental group only. Patients of the amygdala group also increased the reaction speed to positive faces and words with no difference for negative ones and developed a positive bias in the Face-Dot Probe. These results support the possibility to correct the information processing bias in depressed patients via the amygdala upregulation neurofeedback. The authors mention in the discussion that the achieved changes in affective bias are similar to those occurring during pharmacotherapy [148]. Some short-term connectivity changes were detected posttraining. Amygdala links decreased to the temporal pole and increased to some frontal and limbic network areas both at rest and during happy autobiographical memory retrieval. Amygdala-precuneus connectivity was related to self-regulation performance and to clinical outcomes. The study implications included the potential usefulness of the amygdala-precuneus and the amygdala-inferior frontal gyrus connectivity training [149].

Zotev et al. [151] recently presented the data on the protocol of simultaneous upregulation of the left amygdala and the left anterior cingulate fMRI activity along with F3/4 alpha (right over left) and beta (left over right) EEG asymmetry. Sixteen participants with MDD underwent a single session of 4-parameter neurofeedback with a suggested happy autobiographical memory strategy. The session comprised a resting state, a practice run, three main runs, and a transfer run, 8 min and 46 s each run. Main runs included the alternating blocks of rest, neurofeedback, and arithmetical task. Trainees changed both alpha and beta asymmetry, BOLD levels, and connectivity between RoIs during the session with none of these effects in the control group received sham feedback. At the posttraining, participants decreased PoMS depression with *d* = 0.62 and improved on some other mood measures, while controls did not. Alpha asymmetry improvement in the experimental group was negatively correlated with PoMS depression and mood disturbance change and positively linked to MADRS and anhedonia baseline scores suggesting worse is the initial state, more beneficially may be the intervention. The PoMS change intensity was also related to the middle frontal gyrus BOLD laterality in the training. Various neural effects were also discussed, including the changes in EEG asymmetry and left amygdala activity [151]. Further reanalysis of the EEG data with eLORETA showed an asymmetrical shift in a current source density in the prefrontal cortices and in the amygdalae. Shift in alpha distribution was correlated with an anhedonia improvement, while shift in beta band was linked to an anxiety improvement [183].

In one study, female MDD patients were trained to downregulate a functionally defined salience network (voxels within its anatomical borders exhibiting a reaction to negative images from the International Affective Picture System database) response to negative information. Research involved an experimental group and a control group, 10 patients each. Yoked feedback was taken from the data of the trainees from the experimental group. Controls were matched to experimental participants on demographical measures, BDI-II and PANAS scores, and proportion of patients with comorbidity on social anxiety disorder, so randomization was impossible. A single session consisted of three runs (six 34-second trials each). Instruction was to control (diminish) the affective response to a stimulus within the first six seconds after its occurrence. Then, an interstimulus button-pressing task was performed for 16 more seconds. After that, visual feedback on acute affective response was given. Experimental subjects achieved a more prominent decrease in the salience network reactivity to negative stimuli, although connectivity changes of the salience network nodes did not differ between groups. These results were in line with the behavioural changes: the real feedback group showed more improvement on self-evaluated emotional reaction to sad scenes (Cohen's *d* = 0.78) and self-descriptive adjectives (*d* = 0.73), so its participants became less vulnerable. The effect of a single neurofeedback session on depression estimates was not tested [154].

### 5.2. Depressive Disorders Other Than MDD

A recent study [36] sheds light on possible psychological mechanisms of neurofeedback-related improvement in depression. Forty-three medicated participants with a moderate to severe depression having lasted on average more than 10 years by the start of the study were randomized to an experimental group (feedback on a functionally localized area processing positive emotions) and a control group (feedback on the parahippocampal place area); 32 completed the study, and 28 of them were available for a one-month follow-up. It is noteworthy that the sample size was justified based on the expected effect size and required statistical power. The course included three weekly upregulation sessions with feedback and one more on follow-up and also a transfer session after the first two feedback sessions. In both groups, patients successfully upregulated the region of interest in each training session, except the transfer one. The brain activation patterns corresponded to RoI with more anterior insula and striatum activity in the experimental group and more of parahippocampal and lingual gyri response in the control group. A direct contrast revealed a prevalence of a parahippocampal and lingual signal in the control group, but not of an insular in the experimental group. Participants of both groups improved comparably on the HAM-D and on a number of secondary measures including HADS (Hedge's *g* = 1.57 and 2.05 for the experimental and the control group, respectively). Both effect sizes and improvement magnitudes were greater than reported ones for placebo or spontaneous processes and equivalent or greater than were reported for treatment groups in pharmacological, nonpharmacological, and hi-tech treatments for depressions of comparable severity, though such comparisons do not guarantee the specificity of gained effect. Both groups activated the anterior insula to some extent, which could drive some effect; also, the control task could comprise an element of relaxation training (imagery of relaxing sceneries). One of the key findings of the research was that the self-efficacy increase during the course had an inverse relationship with the HAM-D score at the course end point meaning that the self-efficacy gain may be an underlying mechanism of successful depression treatment with neurofeedback.

An interesting proof of concept was published [146] inspired by the Zotev amygdala protocol, but with some important changes. First, a feedback reflected a combination of the bilateral amygdala and hippocampus activity. Second, the picture of a participant's own happy face was presented along with the feedback. Instruction was to retrieve a happy autobiographical memory from the list prepared by each subject in advance and to try feel like in a memory. Thirty-four adolescent patients with different depression diagnoses (mostly MDD and nonspecified depressive disorders; 22 had a comorbid anxiety disorder) and 19 healthy adolescents participated. Participants increased the feedback signal in the neurofeedback runs compared with control ones (counting backward while observing a peer's face). Subsequent analysis showed that these changes were driven by the hippocampi, not the amygdalae. The neurofeedback runs were also associated with more positive emotion rating, and participants improved on performance of recognizing own face posttraining. Depressed participants involved more inferior parietal, cuneus, and fusiform gyrus activity in the neurofeedback, while healthy subjects did not. Unfortunately, the study involved only a single session with a total of 2.5 minutes of neurofeedback condition and no dynamic mood estimate. Right frontal cortex connectivity increased with the right amygdala and decreased with the left amygdala during the neurofeedback, and these changes correlated with depression and rumination score change [184].

Results from this section are summarized in Table 6.

### 5.3. Conclusion

Two most reputable protocols of rt-fMRI NFB have been published to date. The first one targets functionally defined frontal areas involved in positive emotion processing; the suggested strategy is a positive emotional imagery. The second one targets the amygdala and is associated with the strategy of positive autobiographical memories. Both protocols lead to selective fMRI signal changes and clinical improvements. However, the amygdala protocol of Zotev et al. demonstrates specificity of effect against sham neurofeedback (nonemotional brain area regulation), whereas the frontal cortex protocol by Johnston et al. and Linden et al. does not. The number of articles on amygdala neurofeedback in depression is also higher; thus, these studies are rather heterogeneous in terms of the outcome measurement. Functional connectivity changes of the amygdala and other emotional regions after a training course were also reported in studies on left amygdala upregulation protocol, approving the training-driven neuroplasticity changes resulting in a “rewiring” of emotional brain systems. Clinical and behavioural gains in published works are correlated with training-induced functional changes or learning performance which is a strong argument for such neurofeedback validity and its probable recognition in the future.

To date, both protocols for clinical depression meet the criteria of level 2 “possibly efficacious” as studies of each protocol are too heterogeneous. Clinical studies on the frontal protocol were performed in the distinct samples (MDD [152] or milder depressions [36]), while studies of the amygdala protocol were different in terms of the outcome measures (mood in the first study [147], clinical and trait depression measures in the second study [150]). A recent combined fMRI-EEG protocol by Zotev et al. [151] also cannot be treated as equivalent to “pure” fMRI ones.

Other protocols are currently presented in single studies, which allow no solid conclusion about their effectiveness in depression treatment. Formally, the studies on both the left prefrontal cortex [153] and on the salience network [154] neurofeedback in major depression suit level 2 “possibly efficacious.”

## 6. Conclusions

There is no doubt about the role of the neurobiological factors in depression which is reflected in various neuroscientific and biochemical studies; thus, biofeedback and neurofeedback may be reasonable tools to control severity of depression via the underlying biological processes. A priori one could expect more profound evidence for neurofeedback compared to biofeedback in depression with more precise targeting of the known biological substrate (like the amygdala and the prefrontal cortex activity and EEG alpha-band power asymmetry in frontal leads) and increased attention to methodology in the neurofeedback studies of the last decade. However, according to the selected criteria, quite distinct EEG neurofeedback protocols scored equally (namely, level 2 of 5, “possibly efficacious”), even a well-known and widespread in neurofeedback practice alpha frontal asymmetry protocol based on a well-established neural biomarker that matches the results of Larsen and Sherlin [9]. In real-time fMRI neurofeedback, both major protocols also reached only level 2. Among the existing biofeedback modalities, only HRV biofeedback was tested as a treatment of a clinical depression as a primary complication. Studies in this field merit level 3 of 5, “probably efficacious.”

These results do not necessarily mean that neurofeedback is not effective in depression or produces only a nonspecific influence. This review was devoted not directly to the effectiveness of the treatment, but to the existing evidence supporting each protocol in each patient group while each kind of outcome measurement is chosen. So the results of the review are greatly influenced by this aim and the chosen criteria and indicate an absence of proof rather than proof of absence of self-regulation effects in depression.

So why do neurofeedback and biofeedback investigators fail to fulfill their (our) own criteria of evidence collection, and what should be improved in the future studies in the field to strengthen evidence or to confirm the absence of specific effect if it is so? The biofeedback and neurofeedback field seems to have its research traditions determining its strengths and weaknesses. Usually, one of the strong sides is procedure description. Most studies, even those involving rather poor methodology, describe their procedure in all sufficient details or reference to a previously published protocol. On the other hand, we should mention a trend to investigate the effect of combined treatments, e.g., biofeedback+relaxation training or psychoeducation as it was a sole biofeedback intervention that should be sorted out in further studies. Most studies also feature sufficient description of the sample and (yet to lesser degree) all critical information on statistical processing. From the methodological point of view, the majority of controlled studies randomize participants, ensuring baseline equivalence across groups, which is one of the requirements for level 3 of evidence. A rather big portion of controlled studies involve placebo or alternative treatment instead of/along with no treatment/waitlist group, which shows effect specificity and is needed to reach evidence level 4 or 5 in case of positive results.

The first weak point is that the field is overrepresented with pilot trials. Small-sample studies are of use at the onset of a technology or a protocol and are expected when the protocol is documented in one or two papers only, yet these trials are extremely prone to the 2nd type error for low statistical power (requirement of level 2). The absence of a control group does not allow to control for any factor of effect unrelated to treatment, e.g., for spontaneous change of depression severity with the season which bolsters the 1st type error probability. So subsequent studies for each protocol should implement stronger methodology and larger samples and not just multiply proof-of-concept quality data.

The second weak point is a lack of accurate replications. Independent replications are especially needed in the fields of the rt-fMRI neurofeedback (which may be currently prevented by the youth of the technology and low quantity of the research groups conducting the rt-fMRI neurofeedback studies), EEG neurofeedback protocols such as frontal alpha asymmetry, SMR upregulation, and SCP training. They also would be of use for HRV training.

Replications are to be both independent and accurate in terms of (1) critical characteristics of the sample, (2) utilized protocol and biofeedback dosage, (3) control conditions, and (4) outcome measurement. In our vision, for the field of depression treatment, a published study providing results on each of the following outcome groups would have an increased opportunity to be at least partly replicated: (1) psychiatric scale like HAM-D or MADRS; (2) clinically inspired psychological scale targeted specially to measure depression like BDI/BDI-II and HADS and to a lesser degree ZSRDS and SCL-90 depression subscale; (3) mood scale: PANAS or PoMS; (4) some measure of depressive cognitive patterns, e.g., affective bias like the Face-Dot Probe. Other critical parameters of the sample and the procedure, such as medication, group attrition, and side effects, are also to be carefully documented.

## Figures and Tables

**Table 1:** Description of requirements of each level of evidence. Summarized from [11].

Level of evidence	Data supporting clinical efficacy of protocol
(1) Not empirically supported	Anecdotal reports, case studies
(2) Possibly efficacious	One study of sufficient statistical power with well-identified outcome measures; randomization is optional
(3) Probably efficacious	Multiple observational, clinical, waitlist controlled studies; within- and intrasubject replication studies
(4) Efficacious	Two or more independent randomized studies demonstrating superiority of biofeedback to no treatment, alternative treatment or placebo, or noninferiority to treatment as usual (if sufficient statistical power to detect moderate differences is achieved); clearly defined and valid inclusion criteria, treatment procedures, outcome measures, and data analysis
(5) Efficacious and specific	Two or more independent randomized studies demonstrating superiority of biofeedback to sham therapy or alternative treatment

**Table 2:** Summary of the studies in HRV, RSA, and HR biofeedback.

Article	Sample	Demonstrates efficacy	Outcome measures	Statistical power	Control group	Multiple studies	Randomization	Superior to NT, PLA, or AT	Noninferior to TAU	Inclusion criteria	Treatment procedures	Data analysis	2+ independent studies	Superiority to PLA or AT
Required for level		1	2	2	3	3	4	4		4	4	4	4	5
Major depressive disorder (MDD)														
Karavidas et al. [80]	EG = 8	Y	C, P1	Y	—	—	—	—	—	Y	Y	Y	—	—
Caldwell and Steffen [57] (women)	EG = 10 CG = 10	Y	P1	Y	Y	Y	Y	Y	—	Y	Y	Y	Y	—
Breach [81]	EG = 6 CG = 5	Y	C, P1, P2	Y	Y	—	Y	—	—	Y	Y	—	—	—
Lin et al. [82] (sleep problems)	EG = 24 CG = 24	Y	P1	Y	Y	Y	—	Y	—	Y	Y	Y	Y	—
Depressive disorders other than MDD														
Siepmann et al. [83]	EG = 14	Y	P1	Y	—	—	—	—	—	Y	Y	Y	—	—
Bunthumporn [85] (elderly)	EG = 50 CG = 50	Y	P2	Y	Y	—	—	Y	Y	Y	Y	Y	—	—

EG: sample size of the experimental group; CG: sample size of the control group; NT: no treatment; PLA: placebo intervention; AT: alternative treatment; TAU: treatment as usual; Y: yes (criterion fulfilled); C: clinical outcome measure; P1: well-established subjective test of depression; P2: other subjective test of depression.

**Table 3:** Summary of the studies in frontal alpha asymmetry neurofeedback.

Article	Sample	Demonstrates efficacy	Outcome measures	Statistical power	Control group	Multiple studies	Randomization	Superior to NT, PLA, or AT	Noninferior to TAU	Inclusion criteria	Treatment procedures	Data analysis	2+ independent studies	Superiority to PLA or AT
Required for level		1	2	2	3	3	4	4		4	4	4	4	5
Major depressive disorder (MDD)														
Peeters et al. [38]	EG = 9	Y	P2	Y	—	—	—	—	—	Y	Y	Y	—	—
Wang et al. [112]	EG = 24 CG1 = 23 CG2 = 23	Y	P1	Y	Y	—	—	Y	—	Y	Y	Y	—	—
Cheon et al. [113] (F3 *β*↑, Pz *α*↓*θ*↑)	EG = 20	Y	C, P1	Y	—	—	—	—	—	Y	Y	Y	—	—
Depressive disorders other than MDD														
Choi et al. [114]	EG = 12 CG = 11	Y	C, P1, P2	Y	Y	—	Y	Y	—	Y	Y	Y	—	Y/N
Ramirez et al. [115] (Emotiv A↑, *β*/*α*↑, elderly)	EG = 6	Y/N	P1	—	—	—	—	—	—	—	Y	—	—	—

EG: sample size of the experimental group; CG: sample size of the control group; NT: no treatment; PLA: placebo intervention; AT: alternative treatment; TAU: treatment as usual; Y: yes (criterion fulfilled); Y/N: yes/no (criterion partly fulfilled); C: clinical outcome measure; P1: well-established subjective test of depression; P2: other subjective test of depression; A: frontal alpha asymmetry; *α*, *β*, *θ*: EEG frequency bands; ↑: upregulation; ↓: downregulation.

**Table 4:** Summary of the studies in EEG power-based neurofeedback.

Article	Sample	Demonstrates efficacy	Outcome measures	Statistical power	Control group	Multiple studies	Randomization	Superior to NT, PLA, or AT	Noninferior to TAU	Inclusion criteria	Treatment procedures	Data analysis	2+ independent studies	Superiority to PLA or AT
Required for level		1	2	2	3	3	4	4		4	4	4	4	5
Major depressive disorder (MDD)														
Hashemian, Sadjadi [119] (adolescents, F3 *α*/*θ*↓)	EG = 14 CG = 14	Y	C	Y	Y	—	Y	—	—	Y	Y	Y	—	—
Escolano et al. [124] (*α*-2↑)	EG = 40 CG = 20	Y	Cog	Y	Y	—	—	Y	—	Y	Y	Y	—	—
Lee et al. [45] (F3 *β*↑ or T4 SMR↑, Pz *α*↓*θ*↑)	EG = 12 CG = 12	Y	C, P1	Y	Y	—	—	Y	—	Y	Y	Y	—	—
Wang et al. [112] (P3-P4 20-32 Hz↓)	EG = 23 CG1 = 24 CG2 = 23	Y	P1	Y	Y	—	—	Y	—	Y	Y	Y	—	—

EG: sample size of the experimental group; CG: sample size of the control group; NT: no treatment; PLA: placebo intervention; AT: alternative treatment; TAU: treatment as usual; Y: yes (criterion fulfilled); C: clinical outcome measure; P1: well-established subjective test of depression; Cog: cognitive feature of depression; *α*, *β*, *θ*, SMR: EEG frequency bands; ↑: upregulation; ↓: downregulation.

**Table 5:** Summary of the studies in qEEG neurofeedback protocols.

Article	Sample	Demonstrates efficacy	Outcome measures	Statistical power	Control group	Multiple studies	Randomization	Superior to NT, PLA, or AT	Noninferior to TAU	Inclusion criteria	Treatment procedures	Data analysis	2+ independent studies	Superiority to PLA or AT
Required for level		1	2	2	3	3	4	4		4	4	4	4	5
Major depressive disorder (MDD)														
Paquette et al. [140] (qEEG)	EG = 27	Y	C, P1	Y	—	—	—	—	—	Y	Y	Y	—	—

EG: sample size of the experimental group; CG: sample size of the control group; NT: no treatment; PLA: placebo intervention; AT: alternative treatment; TAU: treatment as usual; Y: yes (criterion fulfilled); C: clinical outcome measure; P1: well-established subjective test of depression; qEEG: quantitative EEG.

**Table 6:** Summary of the studies in rt-fMRI neurofeedback.

Article	Sample	Demonstrates efficacy	Outcome measures	Statistical power	Control group	Multiple studies	Randomization	Superior to NT, PLA, or AT	Noninferior to TAU	Inclusion criteria	Treatment procedures	Data analysis	2+ independent studies	Superiority to PLA or AT
Required for level		1	2	2	3	3	4	4		4	4	4	4	5
Major depressive disorder (MDD)														
Linden et al. [152] (positive↑)	EG = 8 CG = 4	Y	C, M	Y/N	Y	—	Y	Y	—	Y	Y	Y	—	Y
Takamura et al. [153] (L DLPFC↑)	EG = 6	Y	C, P1	Y/N	—	—	—	—	—	Y	Y	Y	—	—
Young et al., [147] (L amygdala↑)	EG = 14 CG = 7	Y	M	Y	Y	—	—	Y	—	Y	Y	Y	—	Y
Young et al. [150] (L amygdala↑)	EG = 18 CG = 15	Y	C, P1	Y	Y	—	Y	Y	—	Y	Y	Y	—	Y
Young et al. [148] (L amygdala↑)	EG = 18 CG = 16	Y	Cog	Y	Y	—	Y	Y	—	Y	Y	Y	—	Y
Zotev et al. [151] (L ACC, L amygdala↑, F*α*A, F*β*A)	EG = 16 CG = 8	Y	M	Y	Y	—	—	Y	—	Y	Y	Y	—	Y
Hamilton et al. [154] (salience network↓)	EG = 10 CG = 10	Y	Cog	Y	Y	—	—	Y	—	Y	Y	Y	—	Y
Depressive disorders other than MDD														
Mehler et al. [36] (positive↑)	EG = 16 CG = 16	Y	C, P1	Y	Y	—	Y	Y/N	—	Y	Y	Y	—	—
Quevedo et al. [146] (amygdala+hippocampus↑)	EG = 34	Y	Cog	Y	—	—	—	—	—	Y	Y	Y	—	—

EG: sample size of the experimental group; CG: sample size of the control group; NT: no treatment; PLA: placebo intervention; AT: alternative treatment; TAU: treatment as usual; Y: yes (criterion fulfilled); Y/N: yes/no (criterion partly fulfilled); C: clinical outcome measure; P1: well-established subjective test of depression; M: test of mood; Cog: cognitive feature of depression; Positive: individual regions of interest active while positive vs. neutral stimuli observed; L: left; DLPFC: dorsolateral prefrontal cortex; ACC: anterior cingulate cortex; F*α*A, F*β*A: EEG frontal alpha/beta asymmetry; ↑: upregulation; ↓: downregulation.
